# The Potential Habitat of *Liparis campylostalix* (Orchidaceae) in China Under Climate Change Scenario Predicted by MaxEnt Model

**DOI:** 10.1002/ece3.73536

**Published:** 2026-04-20

**Authors:** Minjie Deng, Yuxuan Zhang, Jiayi Ji, Hua Xu, Bo Qu, Xuhui Chen

**Affiliations:** ^1^ College of Forestry Shenyang Agricultural University Shenyang Liaoning China; ^2^ School of Modern Industry for Selenium Science and Engineering Wuhan Polytechnic University Wuhan China; ^3^ College of Bioscience and Biotechnology Shenyang Agricultural University Shenyang China

**Keywords:** climate change, environment variables, habitat distribution, *Liparis campylostalix*, MaxEnt model, Orchidaceae

## Abstract

Global climate change and human activities have led to the loss and fragmentation of habitats for wild orchids in recent years. *Liparis campylostalix* is widely distributed in northeast China and is an important component of orchids resources in the region. In this study, the MaxEnt model, ENMTools and ArcGIS were utilized to predict the potential habitat of *L. campylostalix* in China based on its geographical distribution and 19 environmental factors, which will be of great significance for the utilization and biodiversity conservation of this species. The results showed that under the current climate scenario, *L. campylostalix* was mainly distributed in Liaoning, Jilin and Anhui provinces of China, with a total suitable growth area of approximately 3.608 million square kilometers and a distribution center located in Sichuan province. The four key environmental variables affecting the distribution of *L. campylostalix* were Precipitation of Warmest Quarter (Bio18), Temperature Seasonality (Bio4), Mean Temperature of Coldest Quarter (Bio11), and Mean Temperature of Driest Quarter (Bio9), accounting for 94.4% of the total contribution. Under global warming conditions in the future, the potential suitable area for *L. campylostalix* would show an expanding trend. It would radiate from the edge of the current suitable area to the northwest region, while the distribution center still located in Sichuan province and not changed. The results of this study could provide theoretical reference for the conservation of orchids biodiversity.

## Graphical abstract

This study used the Maxent model, ENMTools, and ArcGIS to predict *L. campylostalix'*s ecological pattern in China, based on its geographical distribution and environmental factors. Under current climate, it mainly distributes in Liaoning, Jilin, Shandong (suitable area 1.845 million km^2^), with four key variables affecting it: annual precipitation (27.9%), temperature seasonality (19.4%), warmest season precipitation (11.9%), and annual mean temperature (8.4%). Future global warming will expand its potential suitable area (from current edges to surroundings), providing a theoretical reference for orchid biodiversity conservation.

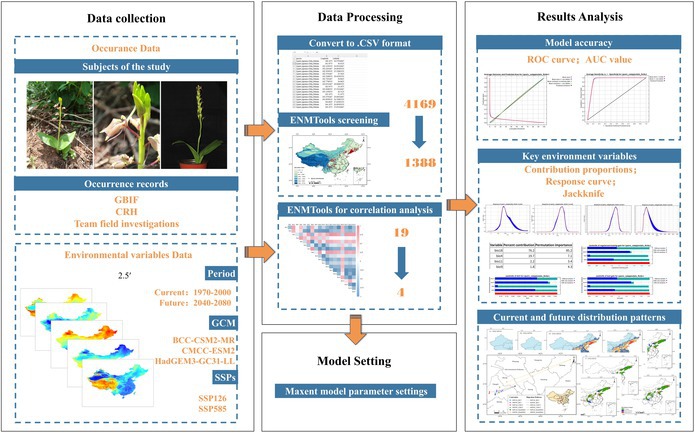

## 1 Introduction

Climate refers to the average weather condition of a region over many years, which is relatively stable and includes factors such as light, temperature, precipitation, and wind power (Nair 2025). These factors together constitute a fundamental environmental space, which is the basis for the survival of organisms on Earth, and determines where organisms will survive, how fast they can grow, how long they can live, and how they reproduce (Thuiller et al. 2005; Clarke and Gaston 2006). Organisms on the Earth have formed diverse physiological, ecological, and evolutionary characteristics in order to adapt to different climatic conditions (Inouye 2022). However, at present, climate change caused by human activities is rapidly changing the average and extreme values of the climate factors, posing severe pressure on the survival of plants and animals that demands our attention (Bell and Collins 2008; Gray and Brady 2016; Inouye 2022).

Due to the characteristic of fixed growth, the survival of plant species is more dependent on the environment (Ramachandran et al. 2025). Accordingly, plants have evolved complex molecular networks to sense and respond to environmental changes (Joseph et al. 2025). For example, secondary metabolites such as proline and soluble sugar can be used as osmotic regulators to help cells maintain water balance under water shortage or freezing conditions (Sharma et al. 2025). Antioxidant enzyme systems such as superoxide dismutase (SOD) and catalase (CAT) are responsible for removing toxic reactive oxygen species produced by abiotic stress, maintaining the stability of intracellular environment and improving plant stress tolerance (García‐Caparrós et al. 2021; Rajput et al. 2021). Late embryogenesis abundant (LEA) can protect cell membrance and protein structure from dehydration damage under drought or low temperature (Amara et al. 2014). Besides, plants could also adjust their life cycle, phenology and the overall morphological structure as a long‐term adaptation strategy (Klisz et al. 2023). It is generally believed that plant growth regulation is a self‐regulation in its basic environmental space, and when the climate changes, plants species first try to stay in their original habitat by adjusting the ontogeny process, and the suitable habitat may not change at this time (Ding et al. 2020; Liu et al. 2023). However, if climate change persists, the ontogeny process will be optimized by the population through intergenerational genetic variation and natural selection, which will eventually lead to migration, expansion or contraction of the suitable habitat areas of the plant species (Jump and Peñuelas 2005; Chuine 2010). The evaluation of potential habitat for plants is helpful to predict the future distribution trend of specific species, so as to provide reference for the rational utilization and management strategies.

Habitat prediction primarily relies on Species Distribution Models (SDMs) combined with species occurrence data and environmental variables (Guisan and Thuiller 2005; Elith and Leathwick 2009). At present, constructed species distribution models mainly include the Maximum Entropy Model (MaxEnt) (Elith et al. 2011), Generalized Linear Model (GLM) (Guisan et al. 2002), Generalized Additive Model (GAM) (Yee and Mitchell 1991), Classification Tree (CT) (Breiman et al. 2017), and Random Forest (RF) (Breiman 2001; Cutler et al. 2007), each applicable to different research purposes and data characteristics. Due to advantages such as convenient operation, high prediction accuracy, and friendliness towards small sample data, MaxEnt has become the preferred model for analyzing key environmental variables and distribution patterns affecting rare and endangered plants (Phillips et al. 2006; Peterson et al. 2007; Warren and Seifert 2011). However, few studies have integrated developmental genetics or plant metabolic regulatory capacity into the SDMs for comprehensive analysis.

**FIGURE 1:**
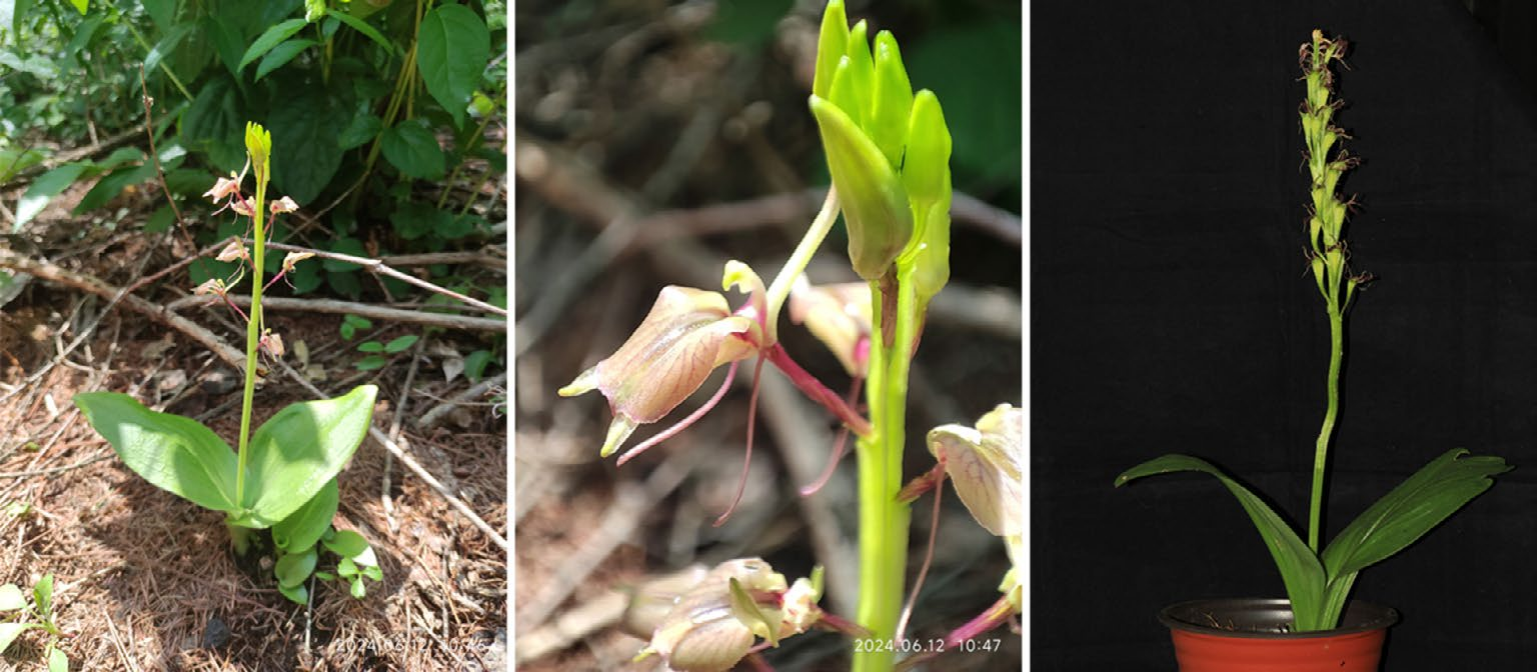
Organism photograph of *L. campylostalix*.

Orchidaceae is one of the largest families of flowering plants and is distributed worldwide. However, many orchid species have suffered dramatic declines in distribution in recent decades, making them a key group of biodiversity conservation (Vitt et al. 2023). *Liparis campylostalix* is a perennial terrestrial orchid species and has a wide geographic distribution in the tropical and subtropical areas such as China, Korea, Japan, and Russia (Flora Republicae Popularis Sinicae Editorial Committee 2004). The plant of *L. campylostalix* is short, with a spherical pseudobulb underground but no obvious aboveground stems and only two ovate leaves in a lifetime. The flowers form racemes, and each flower is yellow green or lavender, with filiform petals and obovate lips (Flora Republicae Popularis Sinicae Editorial Committee 2004) (Figure 1). The pollination mechanism of *L. campylostalix* is still unknown, although it has been reported that the genus *Liparis* could be cross‐pollinated by flies (Adams 1990; Akam 1990) and mosquitoes and could also be self‐pollinated with the assistance of raindrops (Qi et al. 2024). The root is a very thin fibrous root rather than the common fleshy root of orchid species and has the characteristics of symbiosis with *Tulasnella* sp. mycorrhizal fungi (Ding et al. 2014).

Compared with other orchid species in China, *L. campylostalix* has relatively stronger ecological adaptability (Chen et al. 2013). Historical records indicated that *L. campylostalix* had a wide distribution in China, including Heilongjiang, Jilin, Liaoning, Inner Mongolia, Hebei, Shanxi, Shaanxi, Sichuan, and Guizhou provinces (Flora Republicae Popularis Sinicae Editorial Committee 2004). The suitable habitats of *L. campylostalix* mainly include broad‐leaved evergreen forests, pine forests, and the undergrowth of scrub forests, all of which are ecologically suitable for thriving in a diverse array of environmental conditions (Djordjević et al. 2020). Based on the geographic distribution data of *L. campylostalix* and meteorological data from WorldClim, the MaxEnt model and ArcGIS software were utilized in this study to: (1) predict the extent of potential suitable habitat for *L. campylostalix* and changes under various climatic scenarios; (2) investigate the dominant environmental factors influencing the geographic distribution for *L. campylostalix*; (3) forecast the distribution of potential habitat areas and migration routes for *L. campylostalix* in the future. Ultimately, this study aims to establish a scientific foundation for selecting and planning appropriate areas for the conservation of *L. campylostalix* species in China.

## 2 Materials and Methods

### 2.1 Research Framework

**FIGURE 2:**
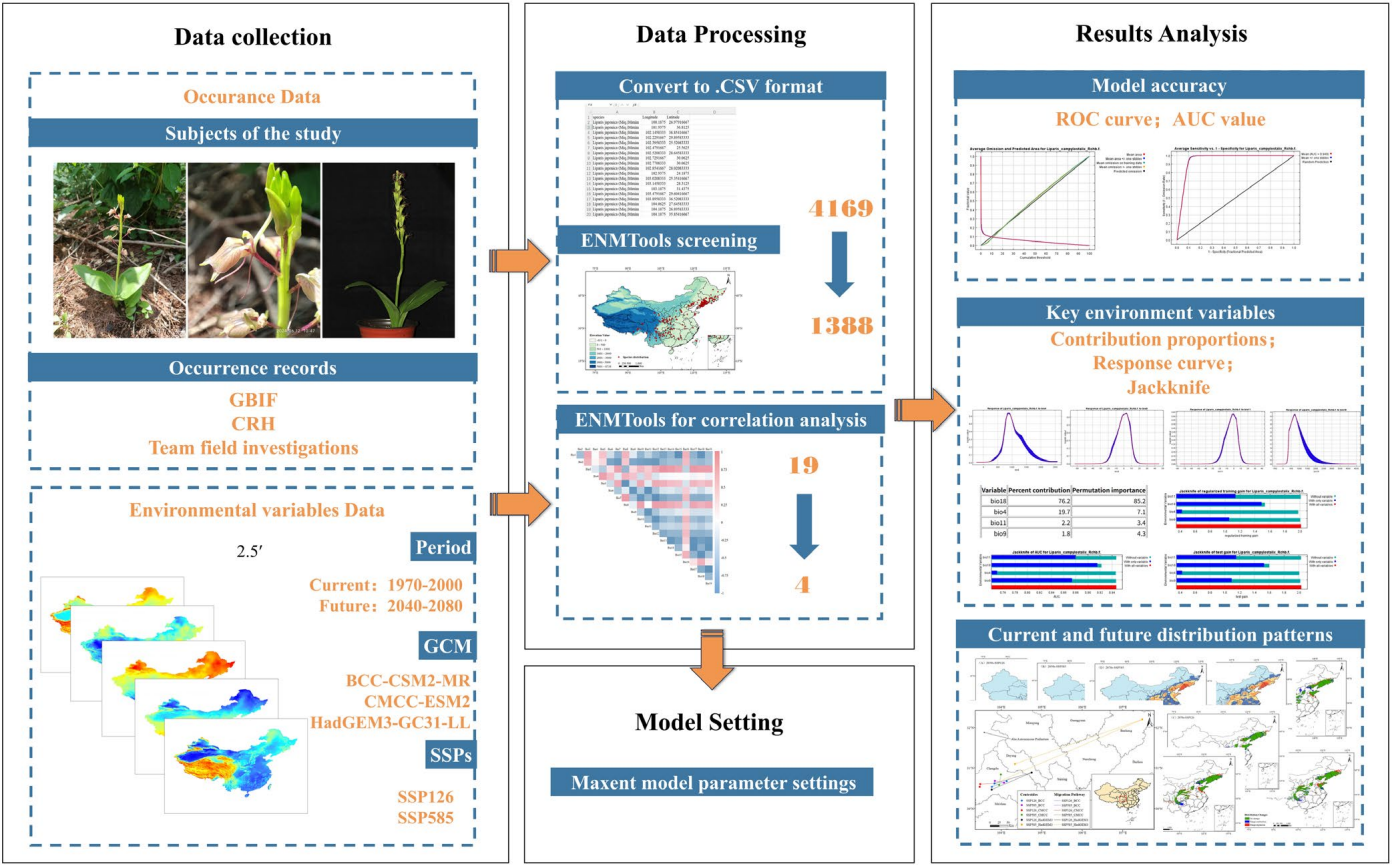
Framework of the contribution of this study.

Based on the research objectives and fundamental principles of the MaxEnt model, a framework was constructed to simulate the current and future suitable distribution of *L. campylostalix* (Figure 2). This framework comprised four components: (1) Data collection: *L. campylostalix* occurrence records and environmental variables were collected. (2) Data processing: The occurrence records were converted to CSV format, filtered to exclude samples that did not conform to the ranges of the environmental variables, and duplicate samples were removed using ENMTools. Environmental variables were resampled to a 2.5‐min resolution, and their redundancy was minimized by analyzing their correlations. (3) Parameter optimization and model setting: The MaxEnt model for *L. campylostalix* was then built and projected to future periods. (4) Simulation and evaluation: Model accuracy was evaluated based on the AUC value. Critical environmental factors were analyzed using contribution proportions, response curves, and jackknife tests. The current and future suitable distributions of *L. campylostalix* were simulated.

### 2.2 Data Collection

#### 2.2.1 Occurrence Data

**FIGURE 3:**
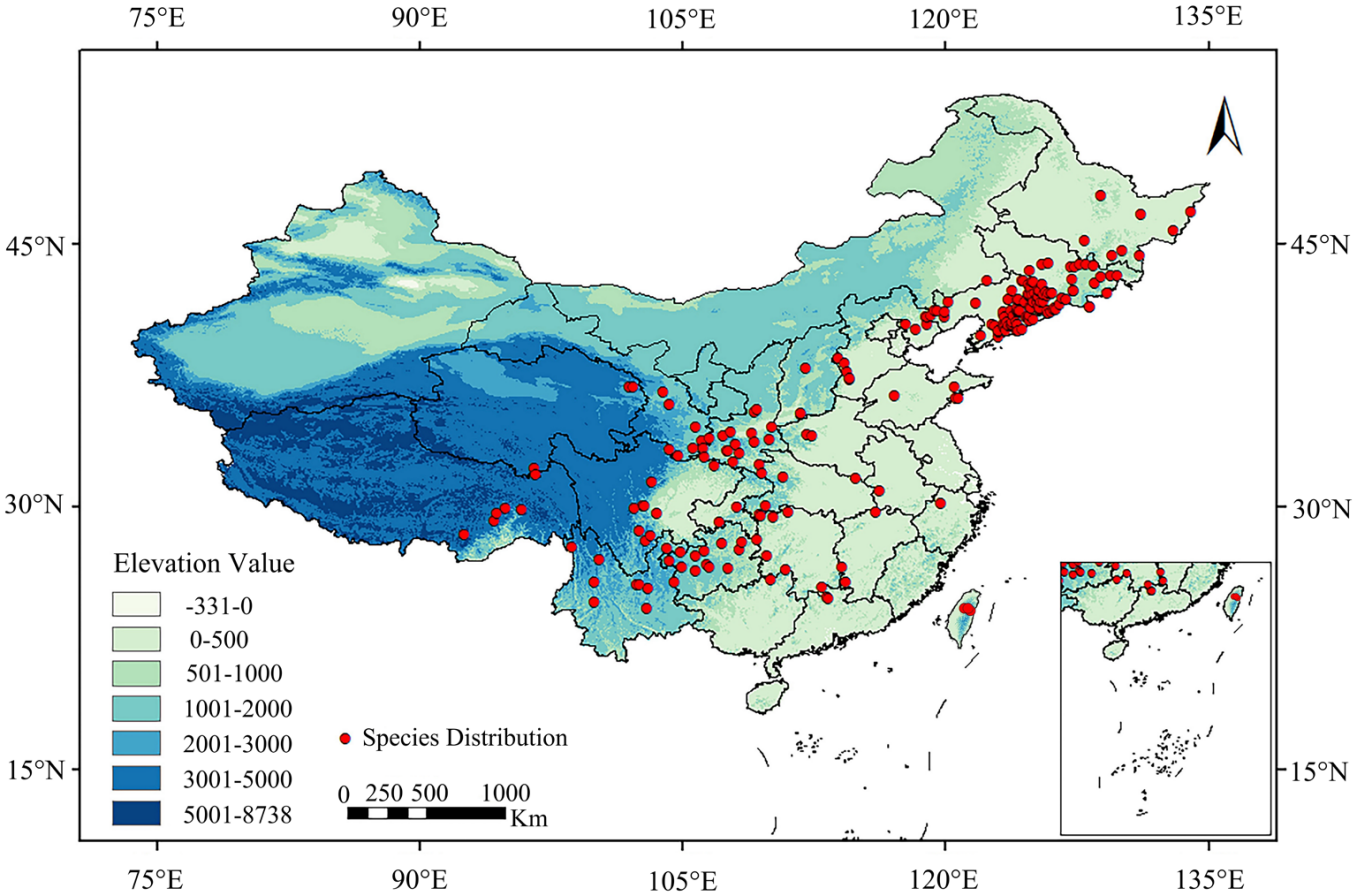
Distribution location of *L. campylostalix* sampling sites.

Distribution data for *L. campylostalix* were primarily obtained through three methods: (1) Geographic point data records of species distribution: A total of 23 distribution points were compiled from the records of *L. campylostalix* in the Chinese Digital Herbarium (https://www.cvh.ac.cn/). (2) GBIF database: A total of 3092 distribution record points for *L. campylostalix* were obtained from the GBIF database (https://www.gbif.org/). (3) Field survey: Our research team collected 1054 distribution points of *L. campylostalix* during the field survey of orchids. After eliminating entries with missing or duplicated latitude and longitude values, as well as those where the coordinates fall in oceanic regions, the three methods produced a total of 4169 records representing the distribution of *L. campylostalix*. To prevent model overfitting caused by the aggregation of distribution points, the ENMTools software platform was employed to screen data (Warren et al. 2010). Only one coordinate point was retained within a 5 km × 5 km raster, resulting in 1388 geographically distributed data points used for simulation (Table S1). Following data processing, the distribution points of *L. campylostalix* in China were extracted with a mask of China, as illustrated in the Figure 3.

#### 2.2.2 Environmental Factors Data

The climate factor data selected for this study consisted of 19 bioclimatic variables obtained from the WorldClim database (Table 1). The WorldClim website provides access to three contemporary climate models (covering the period 1970–2000) and future projections. These future projections include models from the Coupled Model Intercomparison Project Phase 6 (CMIP6): BCC‐CSM2‐MR (Chu et al. 2019), CMCC‐ESM2 (Lovato et al. 2022) and HadGEM3‐GC31‐LL (Andrews et al. 2020). In this study, we compared the periods of the 2050s (2041–2060) and the 2070s (2061–2080). To more efficiently and comprehensively predict the distribution of species habitats under 21st‐century climate change, the selected climate scenarios included a low‐concentration pathway (SSP1‐2.6) and a high‐concentration pathway (SSP5‐8.5). The spatial resolution for each factor was set to 2.5 min.

**TABLE 1:** 19 Environmental factors used in MaxEnt modeling.

Classify	Variables	Description	Units
Temperature	Bio1	Annual mean temperature	°C
	Bio2	Mean diurnal range	°C
	Bio3	Isothermality	—
	Bio4	Temperature seasonality	—
	Bio5	Max temperature of warmest month	°C
	Bio6	Min temperature of coldest month	°C
	Bio7	Temperature annual range	°C
	Bio8	Mean temperature of wettest quarter	°C
	Bio9	Mean temperature of driest quarter	°C
	Bio10	Mean temperature of warmest quarter	°C
	Bio11	Mean temperature of coldest quarter	°C
Precipitation	Bio12	Annual precipitation	mm
	Bio13	Precipitation of wettest month	mm
	Bio14	Precipitation of driest month	mm
	Bio15	Precipitation seasonality	—
	Bio16	Precipitation of wettest quarter	mm
	Bio17	Precipitation of driest quarter	mm
	Bio18	Precipitation of warmest quarter	mm
	Bio19	Precipitation of coldest quarter	mm

### 2.3 Data Selection and Processing

#### 2.3.1 Species Distribution Model Parameter Setting

The geographic distribution point data (CSV format) and the retained bioclimatic variable data (ASC format) for screened *L. campylostalix* were uploaded to MaxEnt version 3.4.4. Receiver Operating Characteristic (ROC) curves were generated to assess the accuracy of the model. The measure of the ROC curve is determined by calculating its area under the curve (AUC) (Melo 2013). The area enclosed by the horizontal and vertical axes of the curve represents the AUC value (Marzban 2004), which ranges from 0 to 1 (Hanley and McNeil 1982). The magnitude of the AUC value directly reflects the strength of the predictive results. Specifically, higher AUC values indicate stronger correlations between environmental variables and the predicted geographical distribution of species, which in turn reflects the robustness of the model's predictive performance (Bradley 1997). In more detail, AUC values ranging from 0.9 to 1.0 indicate excellent predictions, values from 0.8 to 0.9 indicate good predictions, values from 0.7 to 0.8 indicate fair predictions, values from 0.6 to 0.7 indicate unsatisfactory predictions, and values from 0.5 to 0.6 indicate failed predictions (Çorbacıoğlu and Aksel 2023).

#### 2.3.2 Environmental Factors Screening

**FIGURE 4:**
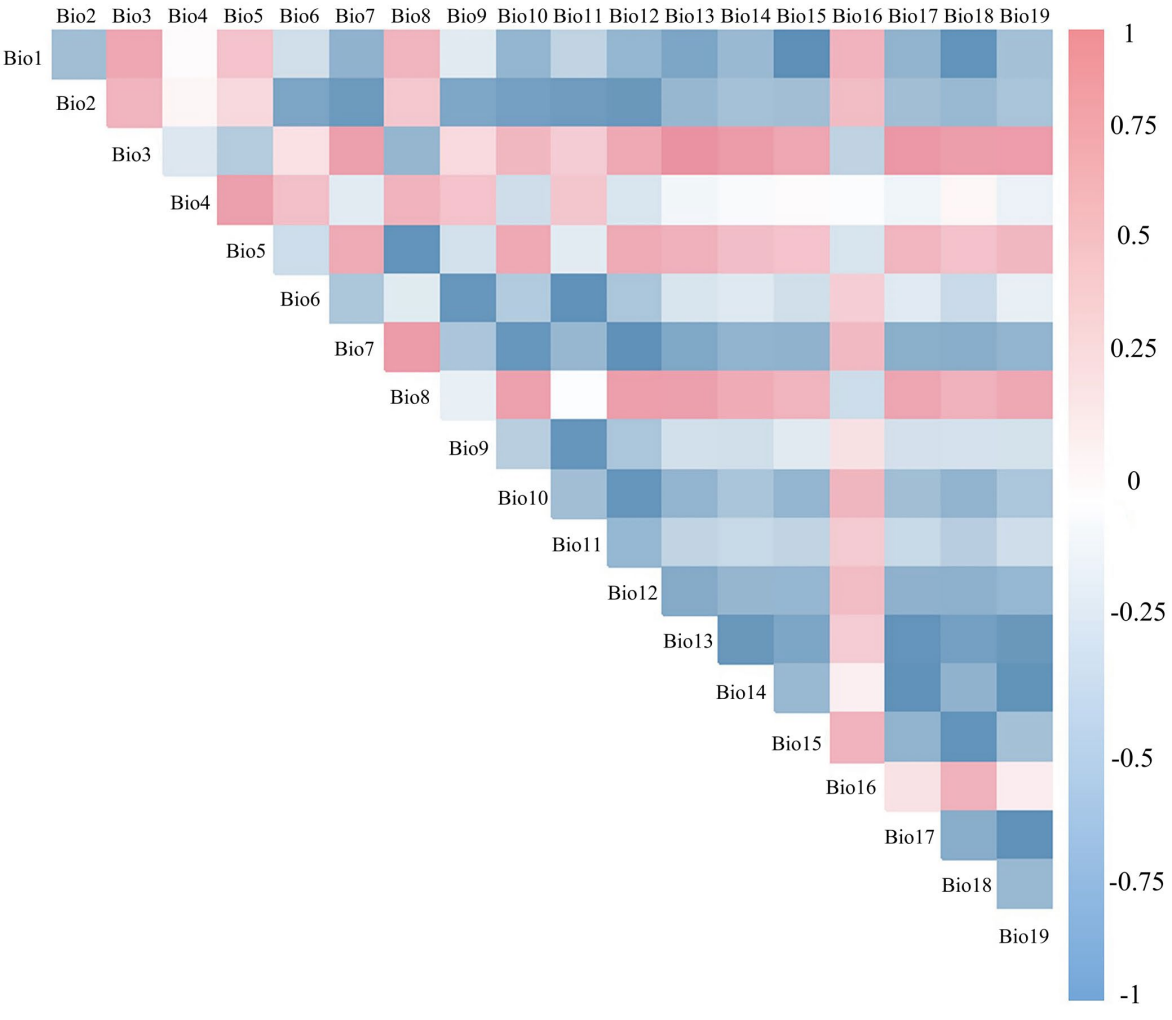
Correlation coefficient matrix of 19 environmental variables.

In order to enhance the accuracy of the simulation and prediction results, MaxEnt software was employed to integrate 19 environmental variables for the initial simulation and prediction of *L. campylostalix* distribution points. On this basis, factors that made no contribution were eliminated. Subsequently, ENMTools software was utilized to perform a Pearson correlation analysis on the remaining factors (Warren et al. 2021) (Figure 4). In the correlation analysis, variables with an absolute correlation coefficient |*r*| less than 0.8 were retained. If |*r*| exceeded 0.8, the variables were re‐evaluated based on their contribution to the model and their relative importance. For the selected key environmental variables, the assessment of importance and the analysis of regularized training gain were conducted. Regularized training gain serves as a quantitative metric for evaluating the specific contribution of each environmental variable to model efficacy (Cobos et al. 2019). This method enables the comparison of the magnitude of their respective impacts by considering the correlations between the variables and normalizing them (Reineking 2006).

### 2.4 Prediction of Potential Suitable Habitats

Suitable habitats were classified into five categories based on probability (*p*) values: extremely unsuitable (*p* ≤ 0.1), unsuitable (0.1 < *p* ≤ 0.2), low suitable (0.2 < *p* ≤ 0.33), moderately suitable (0.33 < *p* ≤ 0.58), and extremely suitable (*p* > 0.58). Data were converted to raster format and reclassified by ArcGIS software. Subsequently, the current and future potential suitable habitat areas were calculated using the spatial analysis module within the same software.

### 2.5 Changes in the Area and Shifts in the Distribution Center of Suitable Habitats for *L. campylostalix*

With the current area of suitable habitat serving as the baseline, the MaxEnt model was utilized to predict suitable habitat distribution under various scenarios from both current and the future. Through this approach, changes in the suitable habitat area for *L. campylostalix* under various scenarios were predicted. Subsequently, the migration trajectories of the distribution center of the habitat layers were traced, thereby enabling the identification of the centroids of suitable habitat distribution for *L. campylostalix* and their migration paths across the various scenarios.

## 3 Results

### 3.1 Model Accuracy Evaluation

**FIGURE 5:**
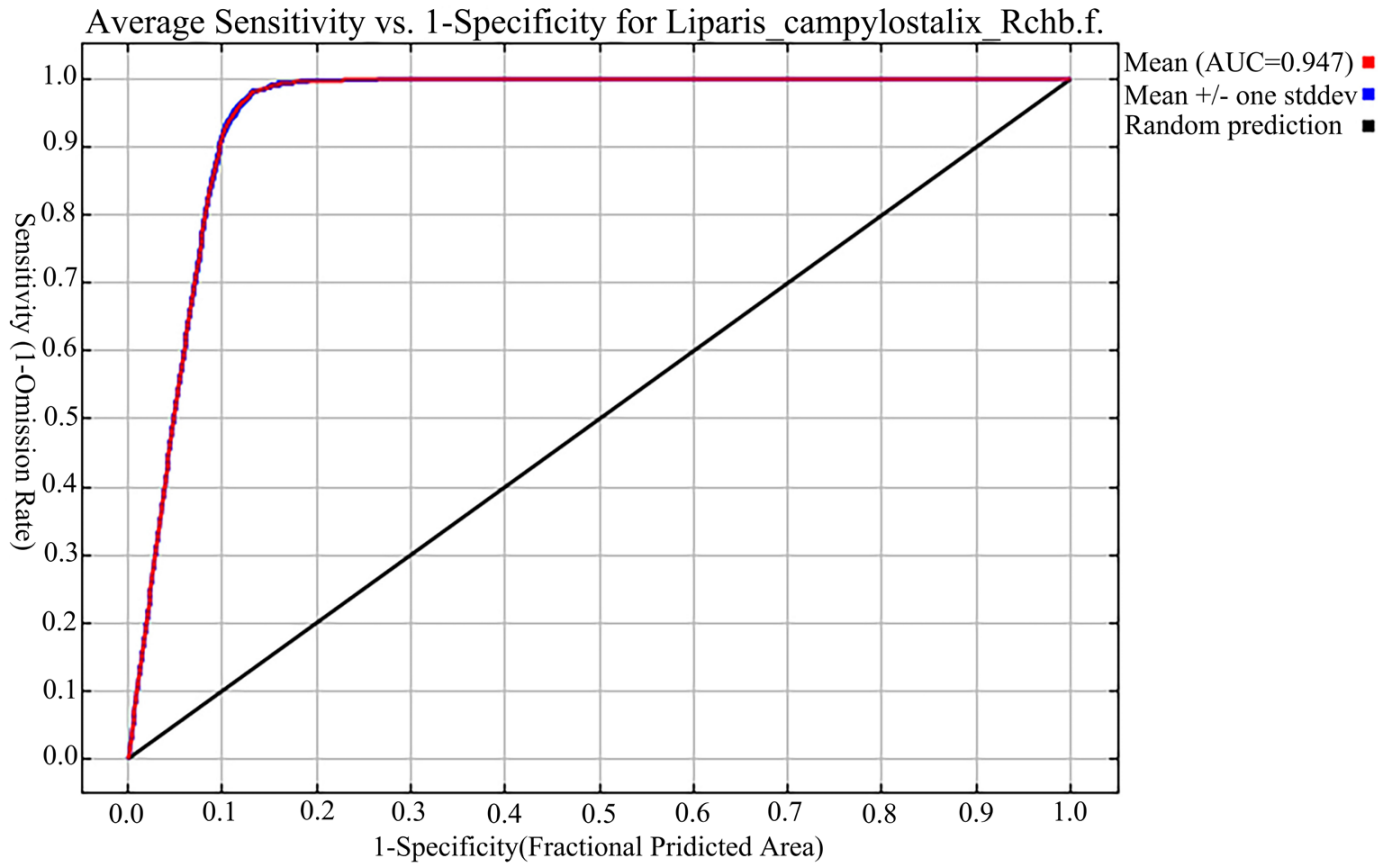
Certification of ROC curve of MaxEnt model on the prediction result of *L. campylostalix*.

In the current scenario, the ROC curve results indicate an AUC value of 0.947 (Figure 5). For the future climate scenarios, the AUC value consistently remained above 0.9 for both the training and test sets. This high‐precision result strongly demonstrates the robustness and reliability of the model in predicting species distributions and provides sufficient support for accurate predictions of suitable habitats for *L. campylostalix*.

### 3.2 Key Environmental Factors Influencing the Distribution of Suitable Habitats for *L. campylostalix*

**FIGURE 6:**
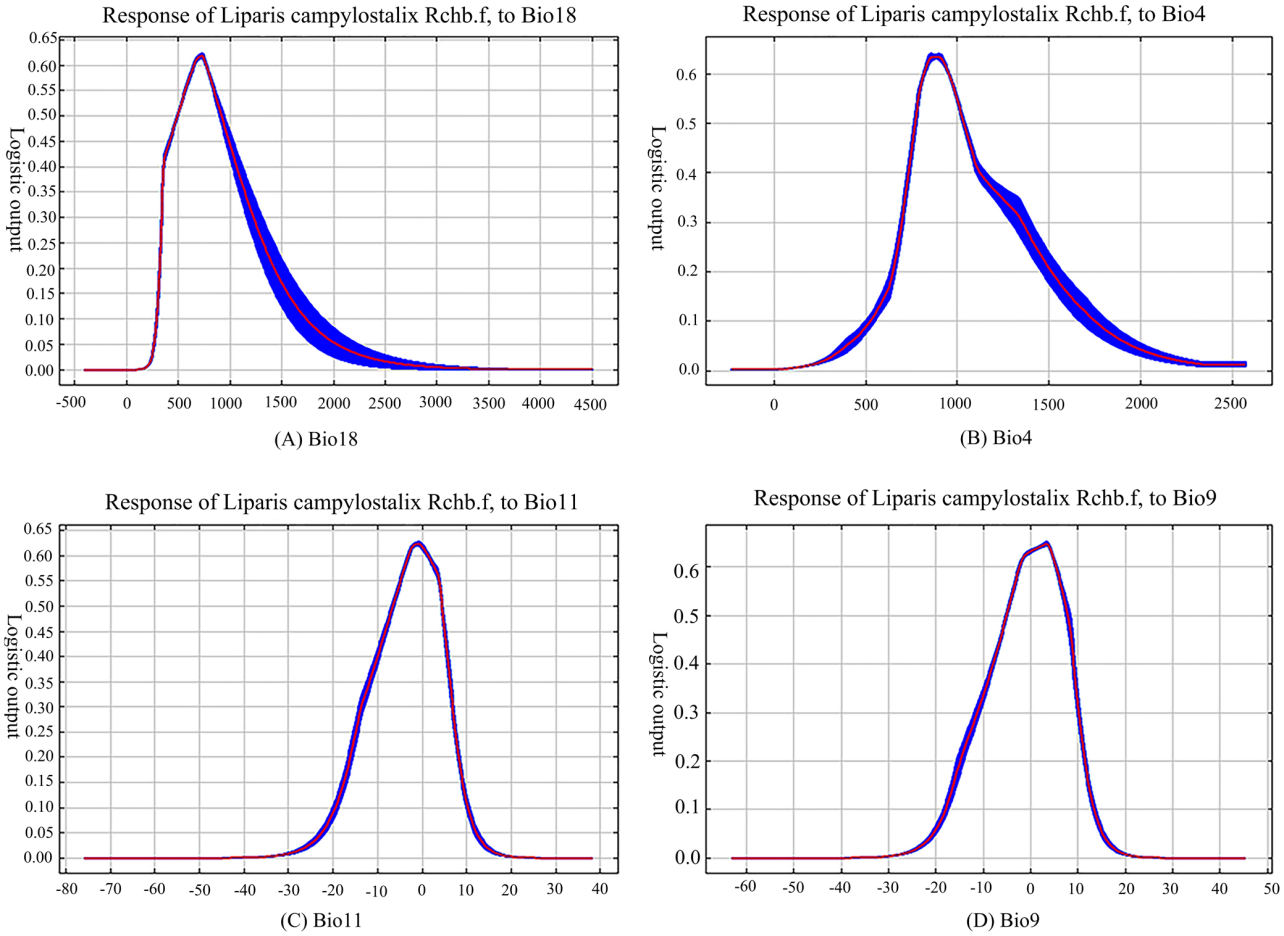
Response curves of four decisive environmental variables. (A) Precipitation of Warmest Quarter (Bio18); (B) Temperature seasonality (Bio4); (C) Mean Temperature of Coldest Quarter (Bio11); (D) Mean Temperature of Driest Quarter (Bio9).

**FIGURE 7:**
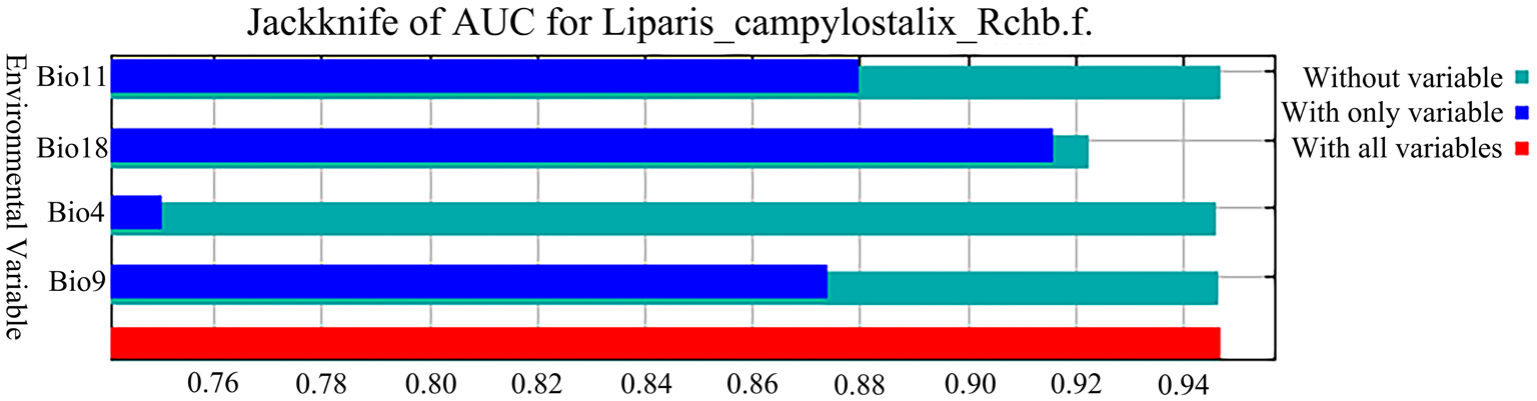
Jackknife test of the MaxEnt model.

The key environmental variables influencing the distribution of *L. campylostalix* were identified by analyzing the percentage contribution of each variable. Initially, MaxEnt was utilized for modeling, and the results indicated that all 19 environmental variables were retained due to their positive contributions in Table 2. According to the results of MaxEnt modeling (Figures 6 and 7), the key environmental variables influencing the distribution of *L. campylostalix* were Precipitation of Warmest Quarter (Bio18), Temperature Seasonality (Bio4), Mean Temperature of Coldest Quarter (Bio11), and Mean Temperature of Driest Quarter (Bio9). Together, these variables accounted for 94.4% of the total contribution, with Bio18 having the most significant impact at 72.8%. This was followed by Bio4, Bio11, and Bio9, which contributed 17.5%, 3.1%, and 1%, respectively. Among the remaining 15 environmental variables, Bio1 and Bio11 exhibited a significant positive correlation; based on the contribution rates of the environmental variables, Bio11 was retained and Bio1 was excluded. The remaining environmental variables had relatively low contribution rates, all below 1%. Among the various environmental factors, temperature‐related variables contributed 21.6%, while precipitation‐related variables accounted for 72.8%. Focusing further on the four climatic factors with the highest contributions (Table 2), the total contribution of precipitation‐related variables was 75.2%, whereas the contribution of temperature‐related variables was 24.7%.

**TABLE 2:** 19 Environmental factors contribution to suitable habitat for *L. campylostalix*.

Classify	Variables	Percent contribution (%)	Permutation importance (%)	Total percent (%)
Temperature	Bio1	1.2	24.8	24.7
	Bio2	0.2	0.1	
	Bio3	0.4	1.8	
	Bio4	17.5	3.9	
	Bio5	0.2	2.1	
	Bio6	0.3	0.2	
	Bio7	0.2	0.5	
	Bio8	0.1	0.8	
	Bio9	1	1.4	
	Bio10	0.5	8.5	
	Bio11	3.1	0	
Precipitation	Bio12	0.2	0.4	75.2
	Bio13	0.9	15.3	
	Bio14	0.1	0.3	
	Bio15	0.9	3.6	
	Bio16	0.2	0.6	
	Bio17	0	0.1	
	Bio18	72.8	35.3	
	Bio19	0.1	0.4	

By examining the response curves of the primary bioclimatic parameters of *L. campylostalix* and their descriptive statistics, we found that the potential distribution probability of *L. campylostalix* peaked at 0.781 when temperature seasonality (Bio4) reached 2343.24, followed by a slight decrease. The ideal temperature seasonality range is from 1812.64 to 2573.44. The optimal range for temperature seasonality was identified as 1391.53 to 2573.44. Temperature seasonality exhibits a monotonically increasing then decreasing trend. The probability of *L. campylostalix* presence increased significantly when mean temperature of coldest quarter (Bio9) ranged from −63.21°C to −12.92°C, peaking at −13.03°C before declining. The optimal temperature range for *L. campylostalix* was determined as −20.20°C to 10.65°C. Mean temperature of coldest quarter shows a fluctuating pattern suggesting a likelihood of gradual increase followed by decrease. The potential distribution probability of *L. campylostalix* began to increase when mean temperature of coldest quarter (Bio11) reached −75.84°C, peaking at 1.23°C. Following this peak, within the temperature range of 1.23°C to 1.35°C, the potential distribution probability showed a gradual decreasing trend. The optimal range for mean temperature of coldest quarter is from −18.38°C to 25.86°C. The potential distribution probability of *L. campylostalix* peaked at 0.614 when precipitation of warmest quarter (Bio18) reached 1594.24 mm, followed by a slight decrease. The ideal precipitation of warmest quarter range is from 479.58 to 4501.20 mm. The probability of *L. campylostalix* presence increased significantly when precipitation of warmest quarter ranged from 268.44 to 4501.20 mm, peaking at 1594.24 mm before declining. The optimal precipitation range for *L. campylostalix* was determined as 366.64 to 4501.20 mm. These results indicate that *L. campylostalix* exhibits strong climatic adaptability, capable of surviving across a wide range of temperatures and precipitation, but with distinct optimal growth condition intervals.

### 3.3 Prediction of Contemporary Potential Habitats for *L. campylostalix*

**FIGURE 8:**
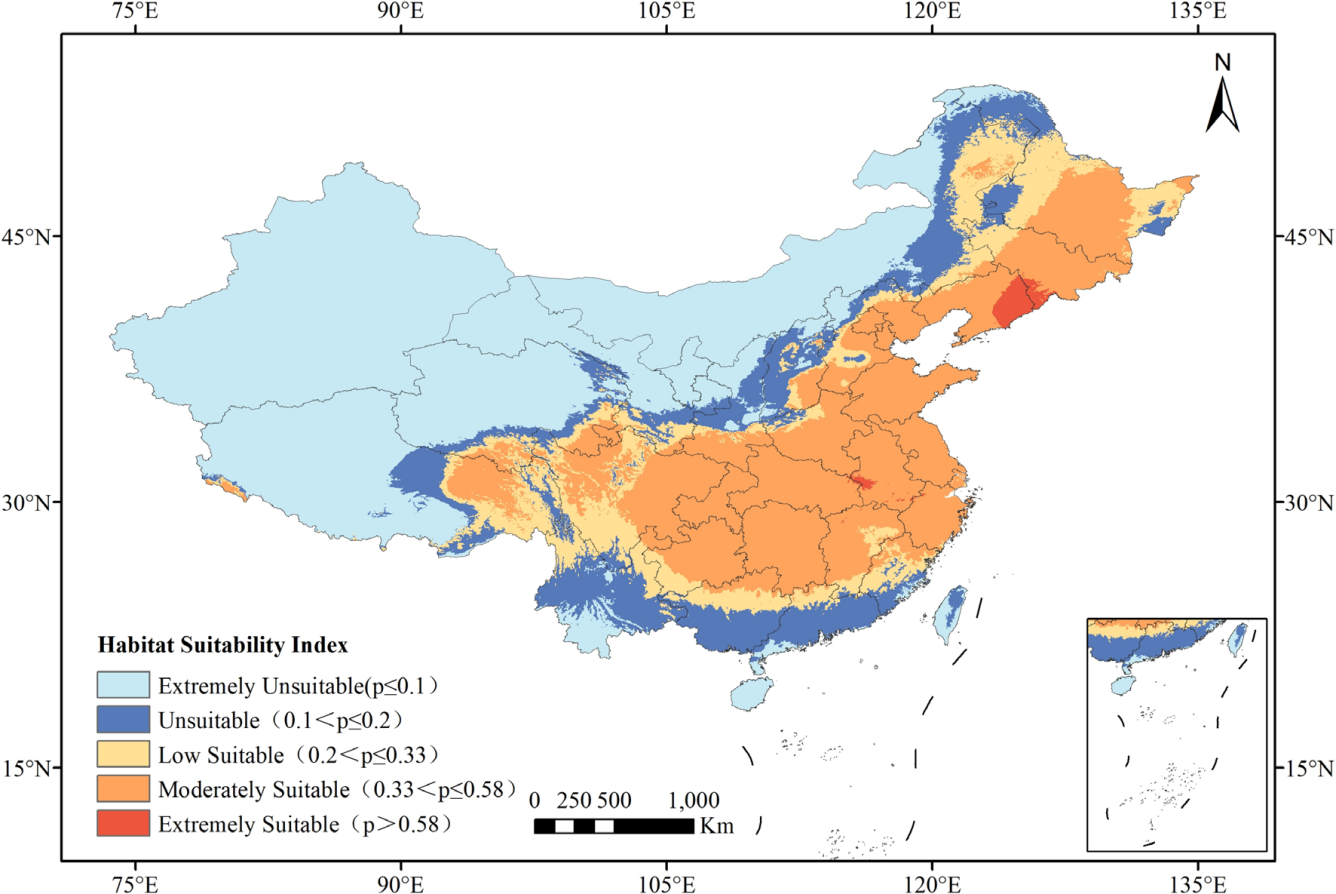
The distribution of suitable growing areas of *L. campylostalix* in China predicted by the MaxEnt model.

The suitable habitats for *L. campylostalix* under the current climate scenario are illustrated in Figure 7. The prediction results indicate that the distribution of *L. campylostalix* at the regional scale in China encompasses several areas, including Liaoning, Jilin, and Shandong, among other provinces. The primary suitable habitats are located in the southeastern coastal provinces of China. The total area of suitable habitats has been calculated and is presented in Figure 8. The overall area suitable for *L. campylostalix* is 360.8 × 10^4^ km^2^, which is equivalent to 37.58% of China's total land area. Among them, the area of highly suitable habitat is 6.55 × 10^4^ km^2^, accounting for 1.81% of the total suitable habitat area. This habitat is distributed in eastern Liaoning, southwestern Jilin, and western Anhui. The area of moderately suitable habitat is 249.83 × 10^4^ km^2^, representing 69.24% of the total suitable habitat area, and is found in the provinces of Heilongjiang, Jilin, Liaoning, Shandong, Henan, Anhui, Jiangsu, Zhejiang, and Hunan, among others. The area of low‐suitability habitat is 104.41 × 10^4^ km^2^, comprising 28.94% of the total suitable habitat area, and is located in northeastern Inner Mongolia, southeastern Sichuan, and other regions. Within China, northwestern regions are not suitable for *L. campylostalix*.

### 3.4 Prediction of Future Potential Habitats for *L. campylostalix*

**FIGURE 9:**
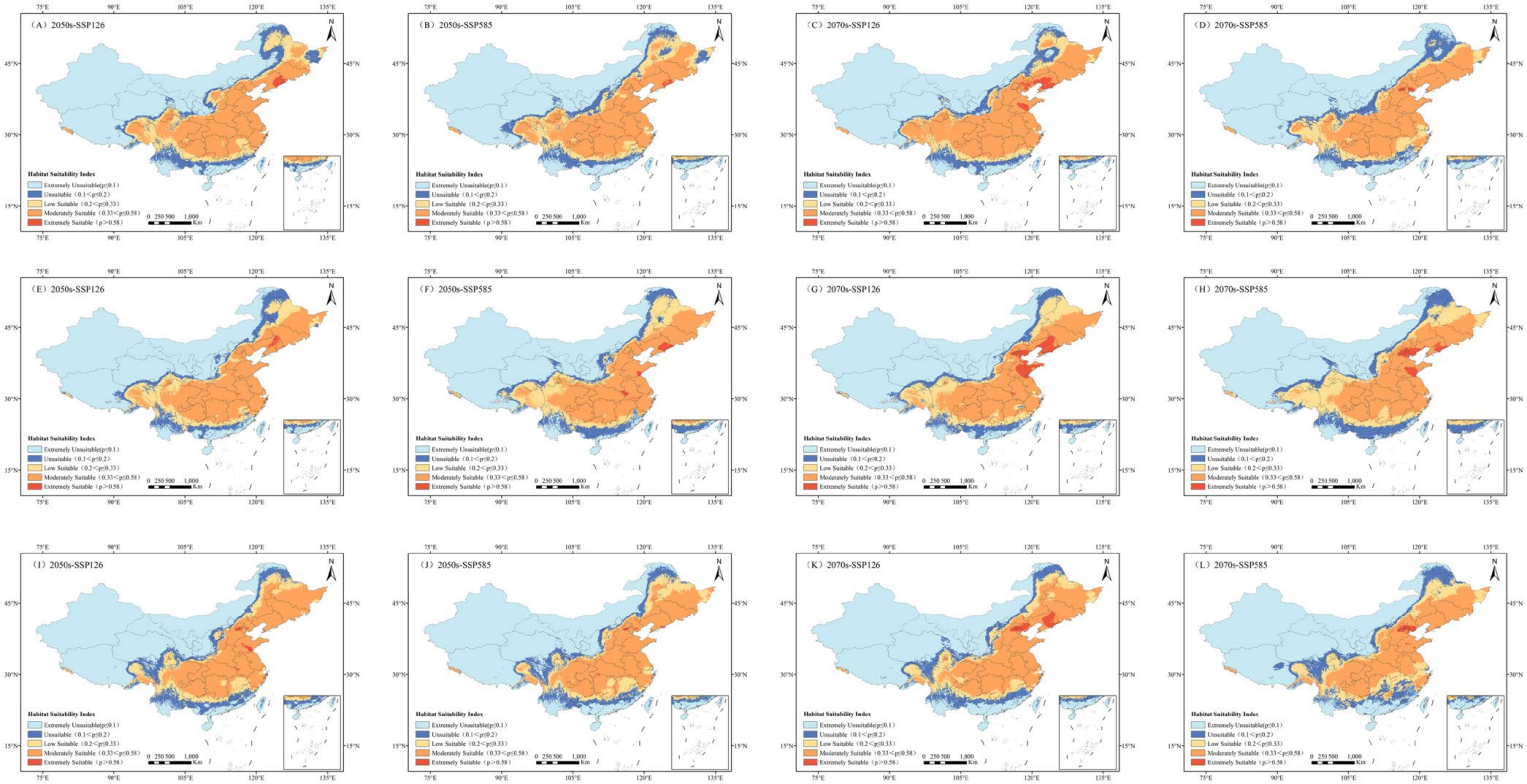
Distribution of suitable areas for *L. campylostalix* in China. (A) BCC‐CSM2‐MR_SSP126_2050s; (B) BCC‐CSM2‐MR_SSP585_2050s; (C) BCC‐CSM2‐MR_SSP126_2070s; (D) BCC‐CSM2‐MR_SSP585_2070s; (E) CMCC‐ESM2_SSP126_2050s; (F) CMCC‐ESM2_SSP585_2050s; (G) CMCC‐ESM2_SSP126_2070s; (H) CMCC‐ESM2_SSP585_2070s; (I) HadGEM3‐GC31‐LL_SSP126_2050s; (J) HadGEM3‐GC31‐LL_SSP585_2050s; (K) HadGEM3‐GC31‐LL_SSP126_2070s; (L) HadGEM3‐GC31‐LL_SSP585_2070s.

**FIGURE 10:**
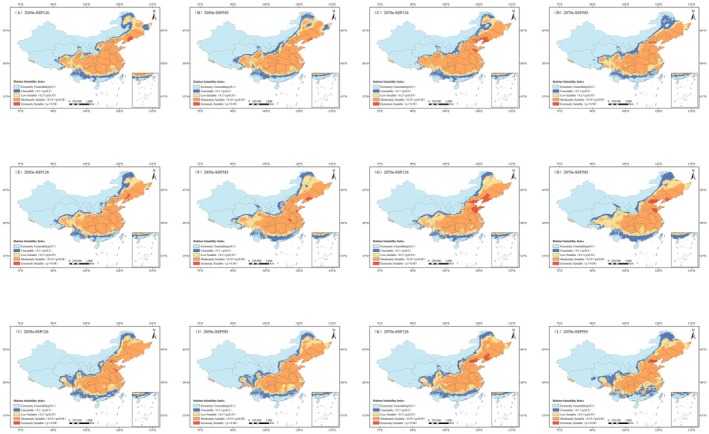
Area and rate of change of suitable habitat for *L. campylostalix* under current and future climate change scenarios.

The MaxEnt model was utilized to predict the potential suitable habitat for *L. campylostalix* under various future climate scenarios. Figure 9 illustrates the predicted spatial distribution pattern of *L. campylostalix* in these scenarios. According to projections by each climate model, the area of potentially suitable habitat for *L. campylostalix* is expected to increase compared to current conditions. This increase is influenced by various socio‐economic development pathways and greenhouse gas emission trajectories. According to the results of the area statistics (Figure 10), most future climate scenarios predict an increase in suitable habitat area. However, the BCC‐CSM2‐MR_SSP585 indicates a decrease in suitable habitat by 4.35 × 10^4^ km^2^ in the 2070s, while the HadGEM3‐GC31‐LL_SSP585 shows a reduction of 11.04 × 10^4^ km^2^ and 8.12 × 10^4^ km^2^ in the 2050s and 2070s respectively.

**FIGURE 11:**
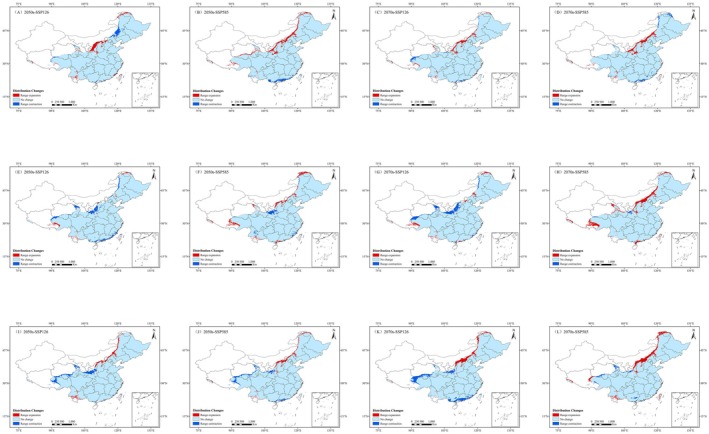
Change on geographical and spatial pattern concerning the suitability area for *L. campylostalix* in contrast with the present. (A) BCC‐CSM2‐MR_SSP126_2050s; (B) BCC‐CSM2‐MR_SSP585_2050s; (C) BCC‐CSM2‐MR_SSP126_2070s; (D) BCC‐CSM2‐MR_SSP585_2070s; (E) CMCC‐ESM2_SSP126_2050s; (F) CMCC‐ESM2_SSP585_2050s; (G) CMCC‐ESM2_SSP126_2070s; (H) CMCC‐ESM2_SSP585_2070s; (I) HadGEM3‐GC31‐LL_SSP126_2050s; (J) HadGEM3‐GC31‐LL_SSP585_2050s; (K) HadGEM3‐GC31‐LL_SSP126_2070s; (L) HadGEM3‐GC31‐LL_SSP585_2070s.

**FIGURE 12:**
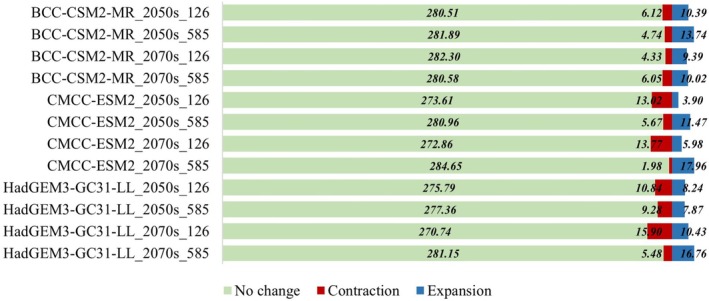
Statistics on the area of change in suitable habitat under different climate models for *L. campylostalix*.

In order to examine the dynamics of suitable areas for *L. campylostalix* under future climate scenarios, current data and future projections were overlaid to generate Figure 11, which was then used to analyze their spatial change patterns. The statistics of contraction and expansion areas are presented in Figure 12. The results of the study indicated that under various future climate scenarios, the suitable habitats for *L. campylostalix* are primarily concentrated in the regions of Liaoning, Jilin, and Anhui, as well as other southeastern regions. Under both pathways, the expansion areas of the distribution range of *L. campylostalix* are primarily concentrated in northern Hebei, northern Shaanxi, and northern Shanxi, with a small‐scale expansion into the low‐suitability areas of the northwest. In contrast, the contraction areas are mostly concentrated in southern Guangdong and southern Guangxi.

### 3.5 Trends in *L. campylostalix* Distribution Center Migration Under Different Climate Scenarios

**FIGURE 13:**
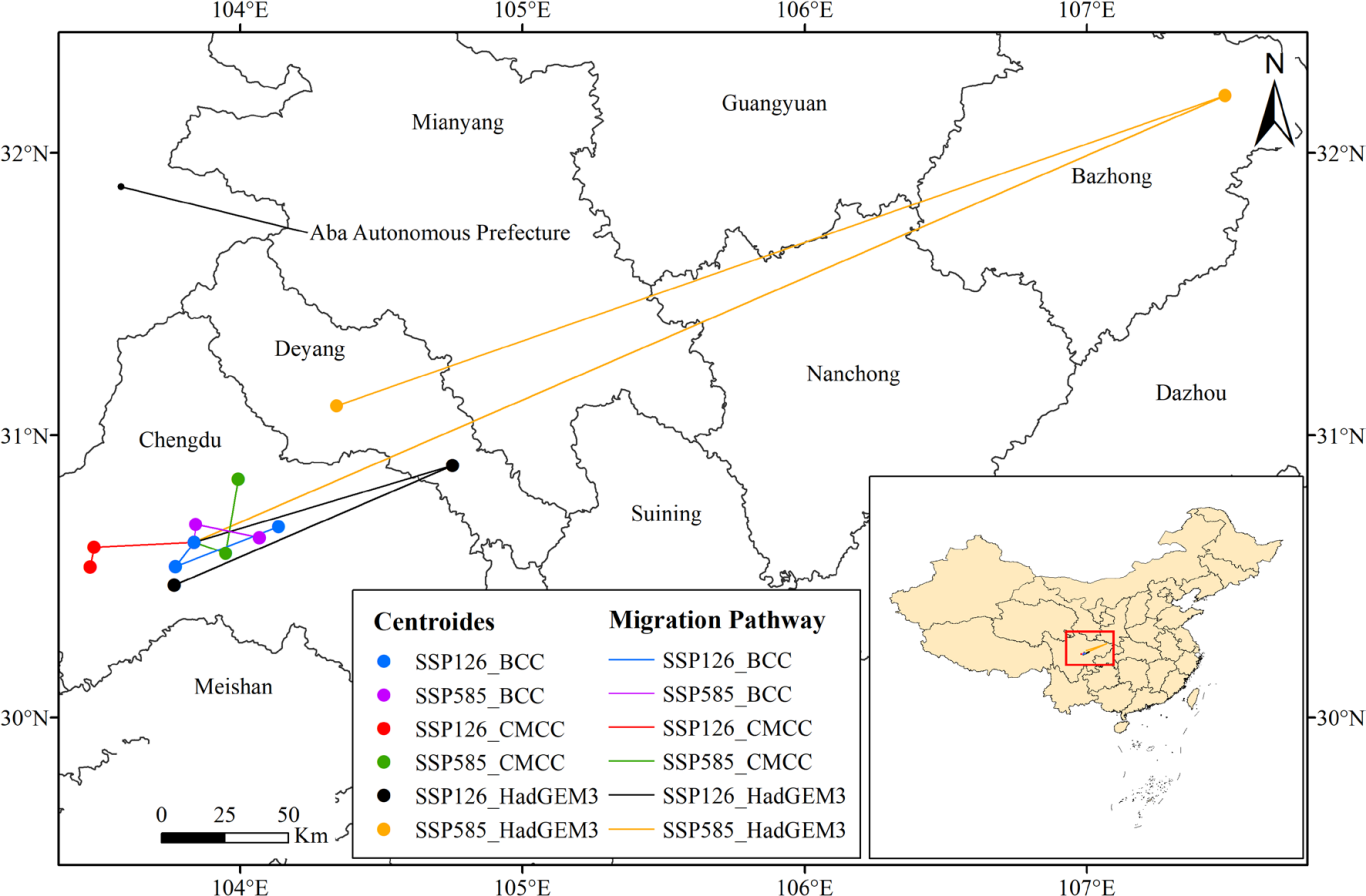
Present distribution of potential habitat and distribution center migration.

Changes in the distribution center migration of *L. campylostalix* under future climate scenarios were illustrated with migration distances detailed in Figure 13 and Table 3. Under contemporary climate conditions, the current center of distribution for *L. campylostalix* is situated in Chengdu City, Sichuan Province (103°50′51″ E, 30°37′41″ N). In predictions of future climate scenarios, the distribution center migration of *L. campylostalix* shows significant variations across different climate models. In the SSP126 scenarios predictions made by the BCC‐CSM2‐MR and CMCC‐ESM2 models, the distribution center of *L. campylostalix* is projected to migrate in a southwesterly direction during the 2050s, with migrations of 13.57 km and 34.89 km, respectively. In the 2070s, the BCC‐CSM2‐MR model forecasts that the distribution center will shift northeastward, while the CMCC‐ESM2 model predicts a continued southward migration. In the SSP585 scenario, the BCC‐CSM2‐MR model predicts a northward migration of the distribution center by 8.54 km in the 2050s, while the CMCC‐ESM2 model forecasts a southward migration. Both models indicate a trend of eastward migration of the distribution center by the 2070s. On the other hand, the HadGEM3‐GC31‐LL model predictions for both the SSP126 and SSP585 scenarios indicate that the distribution center of *L. campylostalix* exhibits a migration trend from the present to the 2070s, moving from the northeast to the southwest.

**TABLE 3:** Distance of the distribution center of *L. campylostalix* under future climate change scenarios.

Future scenarios	2050s (km)	2070s (km)
BCC‐CSM2‐MR_SSP126	13.57	28.95
BCC‐CSM2‐MR_SSP585	8.54	20.09
CMCC‐ESM2_SSP126	34.89	24.60
CMCC‐ESM2_SSP585	10.58	29.16
HadGEM3‐GC31‐LL_SSP126	94.14	19.56
HadGEM3‐GC31‐LL_SSP585	388.07	72.02

## 4 Discussion

### 4.1 Predictive Power of the MaxEnt Model

In this study, we predicted suitable habitats for *L. campylostalix* to enhance our understanding of its distributional characteristics, thereby providing solid scientific support for the application and management of this species. MaxEnt model accuracy was evaluated using the ROC curve, which yielded an AUC value of up to 0.947. AUC values ranging from 0.9 to 1.0 indicate excellent predictive performance (Carter et al. 2016), which suggests that the MaxEnt model has high‐precision forecasting capability. The MaxEnt model is widely recognized for its outstanding prediction accuracy, as supported by numerous studies (Elith et al. 2006; Li and Wang 2013). Species distribution simulations using the MaxEnt model have become a crucial method in conservation ecology, biogeography, and other related fields (Phillips and Dudík 2008).

### 4.2 Key Climatic Factors Restricting the Distribution of *L. campylostalix*

Orchid species are usually sensitive to the environment and their distributions are highly associated with climatic factors, such as elevation, average temperature, solar radiation, precipitation seasonality, precipitation of the warmest quarter, etc. (Gaskett and Gallagher 2018; Ye et al. 2020). In this study, we found that precipitation of warmest quarter (Bio18) played the most important role in the distribution of *L. campylostalix* in China. Accroding to previous studies, precipitation had an important impact on the distribution of many orchid species (Ongaro et al. 2018; De and Biswas 2022). This may be because orchid plants usually have fleshy roots and underdeveloped root hairs, resulting in weak water absorption capacity (Zhang et al. 2018; Lambers and Oliveira 2019). Moreover, precipitation can promote the adaptive changes of root morphology and structure by regulating the cell division speed, cell differentiation direction and the activity of lateral root meristem (Abubakar et al. 2023; Gao, Liu, Zhou, and Zhang 2025; Gao, Liu, Tu, et al. 2025; Gao, Zhang, and Zhang 2025). The roots of *L. campylostalix* is very shallow and underdeveloped. If there is not enough precipitation in the warmest month of the growing season, it may greatly prevent the correct development of the roots (Tatarenko and Kondo 2003). Although *L. campylostalix* has a pseudobulb structure that can store nutrients and water, which can help him get through the bad environment to a certain extent (De and Biswas 2022), the root development determines whether the plant can absorb enough nutrients (Biswas et al. 2021).

### 4.3 Spatial Pattern Changes of *L. campylostalix* Habitat and Distribution Center Migration

Climate change may cause the distribution and survival of some orchid species to be restricted, and thus change the geographical distribution of many orchid species (Ye et al. 2025). Different responses to climate change may lead to the decline or even extinction of some orchid populations with narrow ecological needs (Fay et al. 2025), and may also expand the distribution of other orchid species (Ongaro et al. 2018; Qiu et al. 2023). Our findings showed that the current suitable areas of *L. campylostalix* are expected to remain existing under various climate scenarios in the future, and the potential suitable habitat around the existing area is expected to be significantly expanded. Such results also occurred in *Dactylorhiza hatagirea* and *Eulophia graminea* (Singh et al. 2022; Kolanowska et al. 2024).

The change of temperature and precipitation patterns driven by climate change may be one of the reasons for the increase of the suitable areas of *L. campylostalix*. In fact, in future forecasts, climate change will lead to an increase of the average temperature and precipitation in China, especially in the southwest and northeast regions (Qin et al. 2005), which are just the distribution range of *L. campylostalix*. Combined with our results that precipitation of the warmest quarter is the most important environmental factor affecting the distribution of *L. campylostalix*, it can be suggested that the potential expansion of the suitable areas of *L. campylostalix* may represent a beneficial response to the gradually changing climatic conditions (Ongaro et al. 2018).

The ecological adaptability of plants which are determined by biological characteristics has a deep impact on their response to different climatic conditions (Everingham et al. 2024; Malaviya et al. 2025). Plants could adjust their life cycle, phenology, and the overall morphological structure as a long‐term adaptation strategy (Abubakar et al. 2023; Lang et al. 2025). Based on our study, we think that the strong ecological adaptability of *L. campylostalix* may be another main reason for its continuous expansion of distribution in the future (Chen et al. 2013). *L. campylostalix* can be distributed in many habitats, such as broad‐leaved evergreen forests, pine forests, and the undergrowth of scrub forests, with a large geographical span and great differences in phenological periods (Flora Republicae Popularis Sinicae Editorial Committee 2004), indicating that *L. campylostalix* can flexibly adjust its developmental physiology and phenology to respond to different climatic conditions. Our findings indicated that under various climate change scenarios, the distribution center of *L. campylostalix* in China is located in Sichuan Province, and the distribution center may move to Chengdu, Bazhong, and Deyang in the future, all located in Sichuan Province. The migration pattern of *L. campylostalix* does not conform to the general trend observed in plant species migrating to high latitudes, such as *D. hatagirea* (Singh et al. 2022), which may be attributed to the strong ecological adaptability of *L. campylostalix* (Chen et al. 2013).

However, it is noteworthy that most orchids are highly dependent on specific pollinators for reproduction (Micheneau et al. 2009), and climate‐induced distribution range shifts or phenological mismatches in pollinator populations can severely reduce reproductive success of orchid species (Hutchings et al. 2018; Phillips et al. 2020). Although desynchronization between flowering time and pollinator activity under the global warming situation has rarely been observed so far (Forrest 2015; Schiestl et al. 2025), ignoring the interaction between orchids and pollinators may lead to overestimation of the potential habitat distribution of orchid species (Tsiftsis and Djordjević 2020). The flower of *L. campylostalix* has typical insect pollination characteristics, while its pollinators were not clear so far, which limited the possibility of analysis from the perspective of pollinators. It has been reported that the genus *Liparis* could be cross‐pollinated by flies (Adams 1990; Akam 1990) and mosquitoes (Qi et al. 2024), and could also be self‐pollinated with the assistance of raindrops to ensure successful reproduction (Qi et al. 2024). The pollination mechanism of *L. campylostalix* is worth studying in the future work.

In addition to pollinators, orchids also rely on mycorrhizal fungi for seed germination and nutrient uptake throughout their life cycle (Rasmussen et al. 2015). Climate change can disrupt the distribution and diversity of these fungi and further threaten orchid survival (McCormick and Jacquemyn 2014). Our previous study found that *L. campylostalix* has a relatively specific mycorrhizal symbiosis relationship, mainly with *Tulasnella* fungi (Ding et al. 2016). However, *Tulasnella* fungi are common soil saprophytic fungi which have a wide distribution in different ecological environments (Ding et al. 2014), while *L. campylostalix* is a photosynthetic autotrophic plant which is relatively less dependent on mycorrhizal fungi at the growth stage. Based on this, we speculated that mycorrhizal fungi were not the main factor limiting the distribution of *L. campylostalix*. Saprophytic orchids, which are more dependent on fungal symbiosis, may be more vulnerable to climate change (Swarts and Dixon 2009).

This study dealt only with the effect of climate factors, but future research should also focus on the potential role of its pollinators and mycorrhizal symbionts. By doing this, we will be able to explore whether *L. campylostalix* can shift its distribution under climate change using a more complete spectrum of environmental and biotic constraints. Better insights into the processes affecting the distribution of orchids will be of high value for conservation management and the restoration of orchid populations (Tsiftsis and Djordjević 2020), and the information on the distribution of *L. campylostalix* can serve as a basis for other studies about the potential distribution of other *Liparis* species.

## Author Contributions

**Minjie Deng:** methodology (equal), writing – original draft (equal), writing – review and editing (equal). **Yuxuan Zhang:** formal analysis (equal), validation (equal). **Jiayi Ji:** data curation (equal), formal analysis (equal). **Hua Xu:** investigation (equal), methodology (equal). **Bo Qu:** funding acquisition (equal), investigation (equal), resources (equal), supervision (equal). **Xuhui Chen:** funding acquisition (equal), resources (equal), writing – review and editing (equal).

## Funding

This work was supported by National Natural Science Foundation of China, 31670378 and National Forestry and Grassland Administration, 2021070710.

## Conflicts of Interest

The authors declare no conflicts of interest.

## Supporting information


**Table S1:** The global location sites of *L. campylostalix* used for potential habitat prediction.

## Data Availability

The data that supports the findings of this study are available in the Supporting Information S1.
