# Supplementary material for: The Potential Habitat of Liparis campylostalix (Orchidaceae) in China Under Climate Change Scenario Predicted by MaxEnt Model
Source: Ecol Evol. 2026 Apr 20;16(4):e73536. doi: 10.1002/ece3.73536 (PMC13095647; doi:10.1002/ece3.73536)
Supplement: Supplementary file 1 — Table S1: The global location sites of L. campylostalix used for potential habitat prediction. [file ECE3-16-e73536-s001.docx]

**Table S1** The global location sites of *L. campylostalix* used for potential habitat prediction.

| **Species** | **Latitude** | **Longitude** |
| --- | --- | --- |
| *Liparis campylostalix* Rchb.f. | 21.85417 | 108.0208 |
| *Liparis campylostalix* Rchb.f. | 23.35417 | 103.9375 |
| *Liparis campylostalix* Rchb.f. | 23.8125 | 94.72917 |
| *Liparis campylostalix* Rchb.f. | 23.9375 | 99.27083 |
| *Liparis campylostalix* Rchb.f. | 24.0625 | 121.3958 |
| *Liparis campylostalix* Rchb.f. | 24.1875 | 102.9375 |
| *Liparis campylostalix* Rchb.f. | 24.1875 | 121.0625 |
| *Liparis campylostalix* Rchb.f. | 24.1875 | 121.2708 |
| *Liparis campylostalix* Rchb.f. | 24.4375 | 123.7708 |
| *Liparis campylostalix* Rchb.f. | 24.5625 | 99.9375 |
| *Liparis campylostalix* Rchb.f. | 24.77083 | 113.2708 |
| *Liparis campylostalix* Rchb.f. | 24.8125 | 113.1875 |
| *Liparis campylostalix* Rchb.f. | 24.97917 | 102.6458 |
| *Liparis campylostalix* Rchb.f. | 25.14583 | 108.0208 |
| *Liparis campylostalix* Rchb.f. | 25.35417 | 103.0208 |
| *Liparis campylostalix* Rchb.f. | 25.39583 | 112.9375 |
| *Liparis campylostalix* Rchb.f. | 25.52083 | 102.3958 |
| *Liparis campylostalix* Rchb.f. | 25.5625 | 102.4792 |
| *Liparis campylostalix* Rchb.f. | 25.6875 | 104.4792 |
| *Liparis campylostalix* Rchb.f. | 25.6875 | 114.3125 |
| *Liparis campylostalix* Rchb.f. | 25.6875 | 99.9375 |
| *Liparis campylostalix* Rchb.f. | 25.8125 | 109.9792 |
| *Liparis campylostalix* Rchb.f. | 26.3125 | 105.7292 |
| *Liparis campylostalix* Rchb.f. | 26.4375 | 110.8542 |
| *Liparis campylostalix* Rchb.f. | 26.47917 | 107.5625 |
| *Liparis campylostalix* Rchb.f. | 26.5625 | 104.9375 |
| *Liparis campylostalix* Rchb.f. | 26.5625 | 106.4792 |
| *Liparis campylostalix* Rchb.f. | 26.5625 | 114.1042 |
| *Liparis campylostalix* Rchb.f. | 26.60417 | 127.9792 |
| *Liparis campylostalix* Rchb.f. | 26.64583 | 127.8958 |
| *Liparis campylostalix* Rchb.f. | 26.6875 | 106.3542 |
| *Liparis campylostalix* Rchb.f. | 26.85417 | 102.7708 |
| *Liparis campylostalix* Rchb.f. | 26.85417 | 128.2708 |
| *Liparis campylostalix* Rchb.f. | 26.89583 | 104.1875 |
| *Liparis campylostalix* Rchb.f. | 26.97917 | 100.1875 |
| *Liparis campylostalix* Rchb.f. | 27.1875 | 109.8125 |
| *Liparis campylostalix* Rchb.f. | 27.22917 | 105.7292 |
| *Liparis campylostalix* Rchb.f. | 27.4375 | 104.8542 |
| *Liparis campylostalix* Rchb.f. | 27.4375 | 113.8542 |
| *Liparis campylostalix* Rchb.f. | 27.47917 | 106.2292 |
| *Liparis campylostalix* Rchb.f. | 27.52083 | 108.2292 |
| *Liparis campylostalix* Rchb.f. | 27.64583 | 104.0625 |
| *Liparis campylostalix* Rchb.f. | 27.72917 | 98.64583 |
| *Liparis campylostalix* Rchb.f. | 27.9375 | 107.1875 |
| *Liparis campylostalix* Rchb.f. | 27.97917 | 108.3958 |
| *Liparis campylostalix* Rchb.f. | 28.02083 | 102.8542 |
| *Liparis campylostalix* Rchb.f. | 28.02083 | 98.5625 |
| *Liparis campylostalix* Rchb.f. | 28.14583 | 109.1875 |
| *Liparis campylostalix* Rchb.f. | 28.22917 | 103.9375 |
| *Liparis campylostalix* Rchb.f. | 28.3125 | 103.1458 |
| *Liparis campylostalix* Rchb.f. | 28.4375 | 92.47917 |
| *Liparis campylostalix* Rchb.f. | 28.64583 | 102.5208 |
| *Liparis campylostalix* Rchb.f. | 29.14583 | 107.1042 |
| *Liparis campylostalix* Rchb.f. | 29.22917 | 94.22917 |
| *Liparis campylostalix* Rchb.f. | 29.39583 | 110.1458 |
| *Liparis campylostalix* Rchb.f. | 29.47917 | 109.4375 |
| *Liparis campylostalix* Rchb.f. | 29.52083 | 109.3958 |
| *Liparis campylostalix* Rchb.f. | 29.60417 | 103.4792 |
| *Liparis campylostalix* Rchb.f. | 29.64583 | 94.35417 |
| *Liparis campylostalix* Rchb.f. | 29.6875 | 111.0208 |
| *Liparis campylostalix* Rchb.f. | 29.6875 | 115.9792 |
| *Liparis campylostalix* Rchb.f. | 29.85417 | 95.77083 |
| *Liparis campylostalix* Rchb.f. | 29.89583 | 102.2292 |
| *Liparis campylostalix* Rchb.f. | 29.89583 | 95.52083 |
| *Liparis campylostalix* Rchb.f. | 29.89583 | 95.6875 |
| *Liparis campylostalix* Rchb.f. | 29.9375 | 110.0625 |
| *Liparis campylostalix* Rchb.f. | 29.9375 | 94.8125 |
| *Liparis campylostalix* Rchb.f. | 29.9375 | 94.89583 |
| *Liparis campylostalix* Rchb.f. | 29.97917 | 108.1042 |
| *Liparis campylostalix* Rchb.f. | 30.02083 | 109.7292 |
| *Liparis campylostalix* Rchb.f. | 30.0625 | 102.7292 |
| *Liparis campylostalix* Rchb.f. | 30.0625 | 102.7708 |
| *Liparis campylostalix* Rchb.f. | 30.22917 | 119.7292 |
| *Liparis campylostalix* Rchb.f. | 30.89583 | 116.1875 |
| *Liparis campylostalix* Rchb.f. | 30.97917 | 116.0208 |
| *Liparis campylostalix* Rchb.f. | 31.27083 | 100.7708 |
| *Liparis campylostalix* Rchb.f. | 31.4375 | 103.1875 |
| *Liparis campylostalix* Rchb.f. | 31.5625 | 130.5625 |
| *Liparis campylostalix* Rchb.f. | 31.64583 | 114.8542 |
| *Liparis campylostalix* Rchb.f. | 31.72917 | 110.6875 |
| *Liparis campylostalix* Rchb.f. | 31.85417 | 96.5625 |
| *Liparis campylostalix* Rchb.f. | 31.89583 | 109.5208 |
| *Liparis campylostalix* Rchb.f. | 32.1875 | 96.47917 |
| *Liparis campylostalix* Rchb.f. | 32.27083 | 130.9792 |
| *Liparis campylostalix* Rchb.f. | 32.35417 | 106.8125 |
| *Liparis campylostalix* Rchb.f. | 32.35417 | 130.7708 |
| *Liparis campylostalix* Rchb.f. | 32.39583 | 109.3542 |
| *Liparis campylostalix* Rchb.f. | 32.52083 | 107.8958 |
| *Liparis campylostalix* Rchb.f. | 32.72917 | 130.1875 |
| *Liparis campylostalix* Rchb.f. | 32.8125 | 106.2292 |
| *Liparis campylostalix* Rchb.f. | 32.9375 | 104.6875 |
| *Liparis campylostalix* Rchb.f. | 33.0625 | 108.2292 |
| *Liparis campylostalix* Rchb.f. | 33.14583 | 132.8958 |
| *Liparis campylostalix* Rchb.f. | 33.22917 | 107.5208 |
| *Liparis campylostalix* Rchb.f. | 33.22917 | 107.5625 |
| *Liparis campylostalix* Rchb.f. | 33.22917 | 132.9375 |
| *Liparis campylostalix* Rchb.f. | 33.27083 | 104.2292 |
| *Liparis campylostalix* Rchb.f. | 33.27083 | 126.5208 |
| *Liparis campylostalix* Rchb.f. | 33.27083 | 132.9792 |
| *Liparis campylostalix* Rchb.f. | 33.3125 | 105.6042 |
| *Liparis campylostalix* Rchb.f. | 33.3125 | 106.1458 |
| *Liparis campylostalix* Rchb.f. | 33.3125 | 126.4375 |
| *Liparis campylostalix* Rchb.f. | 33.3125 | 126.4792 |
| *Liparis campylostalix* Rchb.f. | 33.3125 | 126.5208 |
| *Liparis campylostalix* Rchb.f. | 33.3125 | 126.6042 |
| *Liparis campylostalix* Rchb.f. | 33.35417 | 126.3958 |
| *Liparis campylostalix* Rchb.f. | 33.35417 | 126.4375 |
| *Liparis campylostalix* Rchb.f. | 33.35417 | 126.4792 |
| *Liparis campylostalix* Rchb.f. | 33.35417 | 126.5208 |
| *Liparis campylostalix* Rchb.f. | 33.35417 | 126.6458 |
| *Liparis campylostalix* Rchb.f. | 33.35417 | 126.6875 |
| *Liparis campylostalix* Rchb.f. | 33.35417 | 133.0625 |
| *Liparis campylostalix* Rchb.f. | 33.35417 | 133.2292 |
| *Liparis campylostalix* Rchb.f. | 33.35417 | 134.1458 |
| *Liparis campylostalix* Rchb.f. | 33.39583 | 126.3958 |
| *Liparis campylostalix* Rchb.f. | 33.39583 | 126.5625 |
| *Liparis campylostalix* Rchb.f. | 33.39583 | 126.6458 |
| *Liparis campylostalix* Rchb.f. | 33.39583 | 134.1042 |
| *Liparis campylostalix* Rchb.f. | 33.4375 | 126.4792 |
| *Liparis campylostalix* Rchb.f. | 33.4375 | 126.6042 |
| *Liparis campylostalix* Rchb.f. | 33.4375 | 126.6458 |
| *Liparis campylostalix* Rchb.f. | 33.4375 | 133.1042 |
| *Liparis campylostalix* Rchb.f. | 33.4375 | 134.1458 |
| *Liparis campylostalix* Rchb.f. | 33.47917 | 132.8542 |
| *Liparis campylostalix* Rchb.f. | 33.47917 | 133.0208 |
| *Liparis campylostalix* Rchb.f. | 33.47917 | 134.0208 |
| *Liparis campylostalix* Rchb.f. | 33.52083 | 107.9792 |
| *Liparis campylostalix* Rchb.f. | 33.52083 | 133.1042 |
| *Liparis campylostalix* Rchb.f. | 33.52083 | 133.1875 |
| *Liparis campylostalix* Rchb.f. | 33.52083 | 133.8125 |
| *Liparis campylostalix* Rchb.f. | 33.5625 | 108.5208 |
| *Liparis campylostalix* Rchb.f. | 33.5625 | 126.7708 |
| *Liparis campylostalix* Rchb.f. | 33.5625 | 133.0625 |
| *Liparis campylostalix* Rchb.f. | 33.5625 | 133.2292 |
| *Liparis campylostalix* Rchb.f. | 33.5625 | 133.3542 |
| *Liparis campylostalix* Rchb.f. | 33.60417 | 130.4375 |
| *Liparis campylostalix* Rchb.f. | 33.64583 | 133.4375 |
| *Liparis campylostalix* Rchb.f. | 33.64583 | 133.5208 |
| *Liparis campylostalix* Rchb.f. | 33.6875 | 109.1042 |
| *Liparis campylostalix* Rchb.f. | 33.6875 | 132.8125 |
| *Liparis campylostalix* Rchb.f. | 33.6875 | 133.1458 |
| *Liparis campylostalix* Rchb.f. | 33.6875 | 133.3125 |
| *Liparis campylostalix* Rchb.f. | 33.6875 | 133.5208 |
| *Liparis campylostalix* Rchb.f. | 33.6875 | 133.6042 |
| *Liparis campylostalix* Rchb.f. | 33.72917 | 134.0208 |
| *Liparis campylostalix* Rchb.f. | 33.72917 | 134.0625 |
| *Liparis campylostalix* Rchb.f. | 33.77083 | 106.0625 |
| *Liparis campylostalix* Rchb.f. | 33.77083 | 133.1458 |
| *Liparis campylostalix* Rchb.f. | 33.77083 | 134.0208 |
| *Liparis campylostalix* Rchb.f. | 33.77083 | 134.0625 |
| *Liparis campylostalix* Rchb.f. | 33.8125 | 130.9375 |
| *Liparis campylostalix* Rchb.f. | 33.8125 | 133.7708 |
| *Liparis campylostalix* Rchb.f. | 33.8125 | 133.8958 |
| *Liparis campylostalix* Rchb.f. | 33.85417 | 109.9375 |
| *Liparis campylostalix* Rchb.f. | 33.85417 | 133.5625 |
| *Liparis campylostalix* Rchb.f. | 33.89583 | 134.1042 |
| *Liparis campylostalix* Rchb.f. | 33.9375 | 106.5208 |
| *Liparis campylostalix* Rchb.f. | 33.9375 | 133.8542 |
| *Liparis campylostalix* Rchb.f. | 34.0625 | 107.3125 |
| *Liparis campylostalix* Rchb.f. | 34.0625 | 112.3958 |
| *Liparis campylostalix* Rchb.f. | 34.14583 | 112.1042 |
| *Liparis campylostalix* Rchb.f. | 34.14583 | 126.5208 |
| *Liparis campylostalix* Rchb.f. | 34.1875 | 108.9375 |
| *Liparis campylostalix* Rchb.f. | 34.22917 | 135.9375 |
| *Liparis campylostalix* Rchb.f. | 34.27083 | 107.7292 |
| *Liparis campylostalix* Rchb.f. | 34.27083 | 132.3125 |
| *Liparis campylostalix* Rchb.f. | 34.3125 | 126.7708 |
| *Liparis campylostalix* Rchb.f. | 34.39583 | 135.8125 |
| *Liparis campylostalix* Rchb.f. | 34.39583 | 135.8958 |
| *Liparis campylostalix* Rchb.f. | 34.47917 | 135.9375 |
| *Liparis campylostalix* Rchb.f. | 34.52083 | 136.2292 |
| *Liparis campylostalix* Rchb.f. | 34.5625 | 105.7292 |
| *Liparis campylostalix* Rchb.f. | 34.5625 | 110.1042 |
| *Liparis campylostalix* Rchb.f. | 34.5625 | 133.1042 |
| *Liparis campylostalix* Rchb.f. | 34.5625 | 136.2292 |
| *Liparis campylostalix* Rchb.f. | 34.60417 | 127.4375 |
| *Liparis campylostalix* Rchb.f. | 34.64583 | 128.2708 |
| *Liparis campylostalix* Rchb.f. | 34.6875 | 135.8542 |
| *Liparis campylostalix* Rchb.f. | 34.6875 | 135.8958 |
| *Liparis campylostalix* Rchb.f. | 34.72917 | 128.6042 |
| *Liparis campylostalix* Rchb.f. | 34.72917 | 135.2292 |
| *Liparis campylostalix* Rchb.f. | 34.77083 | 127.0208 |
| *Liparis campylostalix* Rchb.f. | 34.77083 | 127.9375 |
| *Liparis campylostalix* Rchb.f. | 34.77083 | 128.6042 |
| *Liparis campylostalix* Rchb.f. | 34.77083 | 133.5208 |
| *Liparis campylostalix* Rchb.f. | 34.77083 | 135.1875 |
| *Liparis campylostalix* Rchb.f. | 34.77083 | 135.2292 |
| *Liparis campylostalix* Rchb.f. | 34.77083 | 135.2708 |
| *Liparis campylostalix* Rchb.f. | 34.8125 | 127.5625 |
| *Liparis campylostalix* Rchb.f. | 34.8125 | 135.1458 |
| *Liparis campylostalix* Rchb.f. | 34.8125 | 135.2292 |
| *Liparis campylostalix* Rchb.f. | 34.8125 | 138.1042 |
| *Liparis campylostalix* Rchb.f. | 34.85417 | 126.8542 |
| *Liparis campylostalix* Rchb.f. | 34.85417 | 127.8542 |
| *Liparis campylostalix* Rchb.f. | 34.85417 | 133.6458 |
| *Liparis campylostalix* Rchb.f. | 34.85417 | 134.5208 |
| *Liparis campylostalix* Rchb.f. | 34.85417 | 137.3542 |
| *Liparis campylostalix* Rchb.f. | 34.85417 | 137.6042 |
| *Liparis campylostalix* Rchb.f. | 34.89583 | 134.6458 |
| *Liparis campylostalix* Rchb.f. | 34.9375 | 127.1042 |
| *Liparis campylostalix* Rchb.f. | 34.9375 | 127.3125 |
| *Liparis campylostalix* Rchb.f. | 34.9375 | 134.0208 |
| *Liparis campylostalix* Rchb.f. | 34.9375 | 135.1042 |
| *Liparis campylostalix* Rchb.f. | 34.9375 | 135.3125 |
| *Liparis campylostalix* Rchb.f. | 34.97917 | 127.8542 |
| *Liparis campylostalix* Rchb.f. | 34.97917 | 133.1875 |
| *Liparis campylostalix* Rchb.f. | 34.97917 | 133.3542 |
| *Liparis campylostalix* Rchb.f. | 34.97917 | 133.9375 |
| *Liparis campylostalix* Rchb.f. | 34.97917 | 135.1875 |
| *Liparis campylostalix* Rchb.f. | 34.97917 | 135.2292 |
| *Liparis campylostalix* Rchb.f. | 34.97917 | 137.8958 |
| *Liparis campylostalix* Rchb.f. | 34.97917 | 138.9375 |
| *Liparis campylostalix* Rchb.f. | 35.02083 | 127.3125 |
| *Liparis campylostalix* Rchb.f. | 35.02083 | 133.6875 |
| *Liparis campylostalix* Rchb.f. | 35.02083 | 134.0208 |
| *Liparis campylostalix* Rchb.f. | 35.02083 | 134.6875 |
| *Liparis campylostalix* Rchb.f. | 35.02083 | 135.2292 |
| *Liparis campylostalix* Rchb.f. | 35.0625 | 127.3958 |
| *Liparis campylostalix* Rchb.f. | 35.0625 | 134.4792 |
| *Liparis campylostalix* Rchb.f. | 35.10417 | 127.3542 |
| *Liparis campylostalix* Rchb.f. | 35.10417 | 127.5625 |
| *Liparis campylostalix* Rchb.f. | 35.10417 | 133.8958 |
| *Liparis campylostalix* Rchb.f. | 35.10417 | 134.3542 |
| *Liparis campylostalix* Rchb.f. | 35.10417 | 134.4375 |
| *Liparis campylostalix* Rchb.f. | 35.10417 | 135.2708 |
| *Liparis campylostalix* Rchb.f. | 35.10417 | 135.7708 |
| *Liparis campylostalix* Rchb.f. | 35.10417 | 137.3125 |
| *Liparis campylostalix* Rchb.f. | 35.10417 | 137.3542 |
| *Liparis campylostalix* Rchb.f. | 35.10417 | 139.0625 |
| *Liparis campylostalix* Rchb.f. | 35.10417 | 140.1875 |
| *Liparis campylostalix* Rchb.f. | 35.14583 | 127.1458 |
| *Liparis campylostalix* Rchb.f. | 35.14583 | 127.3958 |
| *Liparis campylostalix* Rchb.f. | 35.14583 | 133.5625 |
| *Liparis campylostalix* Rchb.f. | 35.14583 | 133.8125 |
| *Liparis campylostalix* Rchb.f. | 35.14583 | 134.1458 |
| *Liparis campylostalix* Rchb.f. | 35.14583 | 137.4375 |
| *Liparis campylostalix* Rchb.f. | 35.14583 | 137.7292 |
| *Liparis campylostalix* Rchb.f. | 35.14583 | 138.6042 |
| *Liparis campylostalix* Rchb.f. | 35.14583 | 139.0208 |
| *Liparis campylostalix* Rchb.f. | 35.14583 | 139.0625 |
| *Liparis campylostalix* Rchb.f. | 35.14583 | 139.1042 |
| *Liparis campylostalix* Rchb.f. | 35.1875 | 127.1458 |
| *Liparis campylostalix* Rchb.f. | 35.1875 | 127.6875 |
| *Liparis campylostalix* Rchb.f. | 35.1875 | 129.1458 |
| *Liparis campylostalix* Rchb.f. | 35.1875 | 133.4375 |
| *Liparis campylostalix* Rchb.f. | 35.1875 | 133.5625 |
| *Liparis campylostalix* Rchb.f. | 35.1875 | 133.6042 |
| *Liparis campylostalix* Rchb.f. | 35.1875 | 133.9792 |
| *Liparis campylostalix* Rchb.f. | 35.1875 | 134.0625 |
| *Liparis campylostalix* Rchb.f. | 35.1875 | 134.1875 |
| *Liparis campylostalix* Rchb.f. | 35.1875 | 134.2708 |
| *Liparis campylostalix* Rchb.f. | 35.1875 | 134.3958 |
| *Liparis campylostalix* Rchb.f. | 35.1875 | 134.8125 |
| *Liparis campylostalix* Rchb.f. | 35.1875 | 137.2708 |
| *Liparis campylostalix* Rchb.f. | 35.1875 | 139.0208 |
| *Liparis campylostalix* Rchb.f. | 35.1875 | 139.0625 |
| *Liparis campylostalix* Rchb.f. | 35.1875 | 139.1042 |
| *Liparis campylostalix* Rchb.f. | 35.1875 | 140.1042 |
| *Liparis campylostalix* Rchb.f. | 35.22917 | 133.9375 |
| *Liparis campylostalix* Rchb.f. | 35.22917 | 134.4375 |
| *Liparis campylostalix* Rchb.f. | 35.22917 | 134.4792 |
| *Liparis campylostalix* Rchb.f. | 35.22917 | 134.5208 |
| *Liparis campylostalix* Rchb.f. | 35.22917 | 134.6458 |
| *Liparis campylostalix* Rchb.f. | 35.22917 | 134.9375 |
| *Liparis campylostalix* Rchb.f. | 35.22917 | 137.4792 |
| *Liparis campylostalix* Rchb.f. | 35.22917 | 137.6458 |
| *Liparis campylostalix* Rchb.f. | 35.22917 | 137.6875 |
| *Liparis campylostalix* Rchb.f. | 35.22917 | 137.8125 |
| *Liparis campylostalix* Rchb.f. | 35.22917 | 137.8542 |
| *Liparis campylostalix* Rchb.f. | 35.22917 | 138.9792 |
| *Liparis campylostalix* Rchb.f. | 35.22917 | 139.0208 |
| *Liparis campylostalix* Rchb.f. | 35.22917 | 139.0625 |
| *Liparis campylostalix* Rchb.f. | 35.27083 | 111.6875 |
| *Liparis campylostalix* Rchb.f. | 35.27083 | 127.6042 |
| *Liparis campylostalix* Rchb.f. | 35.27083 | 129.0625 |
| *Liparis campylostalix* Rchb.f. | 35.27083 | 133.8125 |
| *Liparis campylostalix* Rchb.f. | 35.27083 | 134.1042 |
| *Liparis campylostalix* Rchb.f. | 35.27083 | 134.5625 |
| *Liparis campylostalix* Rchb.f. | 35.27083 | 134.9375 |
| *Liparis campylostalix* Rchb.f. | 35.27083 | 135.0208 |
| *Liparis campylostalix* Rchb.f. | 35.27083 | 137.6042 |
| *Liparis campylostalix* Rchb.f. | 35.27083 | 137.6875 |
| *Liparis campylostalix* Rchb.f. | 35.27083 | 137.7292 |
| *Liparis campylostalix* Rchb.f. | 35.27083 | 137.8542 |
| *Liparis campylostalix* Rchb.f. | 35.27083 | 138.7708 |
| *Liparis campylostalix* Rchb.f. | 35.27083 | 139.0208 |
| *Liparis campylostalix* Rchb.f. | 35.27083 | 139.0625 |
| *Liparis campylostalix* Rchb.f. | 35.3125 | 111.6875 |
| *Liparis campylostalix* Rchb.f. | 35.3125 | 127.1042 |
| *Liparis campylostalix* Rchb.f. | 35.3125 | 127.5208 |
| *Liparis campylostalix* Rchb.f. | 35.3125 | 127.8542 |
| *Liparis campylostalix* Rchb.f. | 35.3125 | 134.5208 |
| *Liparis campylostalix* Rchb.f. | 35.3125 | 134.6042 |
| *Liparis campylostalix* Rchb.f. | 35.3125 | 135.6042 |
| *Liparis campylostalix* Rchb.f. | 35.3125 | 137.8542 |
| *Liparis campylostalix* Rchb.f. | 35.3125 | 137.9792 |
| *Liparis campylostalix* Rchb.f. | 35.3125 | 138.3542 |
| *Liparis campylostalix* Rchb.f. | 35.3125 | 138.6875 |
| *Liparis campylostalix* Rchb.f. | 35.3125 | 139.0208 |
| *Liparis campylostalix* Rchb.f. | 35.3125 | 139.0625 |
| *Liparis campylostalix* Rchb.f. | 35.3125 | 139.1042 |
| *Liparis campylostalix* Rchb.f. | 35.3125 | 139.6042 |
| *Liparis campylostalix* Rchb.f. | 35.35417 | 127.6042 |
| *Liparis campylostalix* Rchb.f. | 35.35417 | 127.6458 |
| *Liparis campylostalix* Rchb.f. | 35.35417 | 127.6875 |
| *Liparis campylostalix* Rchb.f. | 35.35417 | 127.7292 |
| *Liparis campylostalix* Rchb.f. | 35.35417 | 127.8542 |
| *Liparis campylostalix* Rchb.f. | 35.35417 | 127.8958 |
| *Liparis campylostalix* Rchb.f. | 35.35417 | 128.0208 |
| *Liparis campylostalix* Rchb.f. | 35.35417 | 133.6042 |
| *Liparis campylostalix* Rchb.f. | 35.35417 | 134.0625 |
| *Liparis campylostalix* Rchb.f. | 35.35417 | 134.5208 |
| *Liparis campylostalix* Rchb.f. | 35.35417 | 137.7292 |
| *Liparis campylostalix* Rchb.f. | 35.35417 | 137.8125 |
| *Liparis campylostalix* Rchb.f. | 35.35417 | 137.9375 |
| *Liparis campylostalix* Rchb.f. | 35.39583 | 109.1042 |
| *Liparis campylostalix* Rchb.f. | 35.39583 | 126.9792 |
| *Liparis campylostalix* Rchb.f. | 35.39583 | 127.9375 |
| *Liparis campylostalix* Rchb.f. | 35.39583 | 128.0625 |
| *Liparis campylostalix* Rchb.f. | 35.39583 | 134.4375 |
| *Liparis campylostalix* Rchb.f. | 35.39583 | 137.6875 |
| *Liparis campylostalix* Rchb.f. | 35.39583 | 137.7292 |
| *Liparis campylostalix* Rchb.f. | 35.39583 | 137.9375 |
| *Liparis campylostalix* Rchb.f. | 35.39583 | 138.5625 |
| *Liparis campylostalix* Rchb.f. | 35.39583 | 138.6875 |
| *Liparis campylostalix* Rchb.f. | 35.39583 | 138.8125 |
| *Liparis campylostalix* Rchb.f. | 35.39583 | 138.8958 |
| *Liparis campylostalix* Rchb.f. | 35.39583 | 138.9375 |
| *Liparis campylostalix* Rchb.f. | 35.39583 | 139.0625 |
| *Liparis campylostalix* Rchb.f. | 35.4375 | 126.7292 |
| *Liparis campylostalix* Rchb.f. | 35.4375 | 126.8958 |
| *Liparis campylostalix* Rchb.f. | 35.4375 | 127.3125 |
| *Liparis campylostalix* Rchb.f. | 35.4375 | 127.6875 |
| *Liparis campylostalix* Rchb.f. | 35.4375 | 127.8958 |
| *Liparis campylostalix* Rchb.f. | 35.4375 | 127.9792 |
| *Liparis campylostalix* Rchb.f. | 35.4375 | 128.0625 |
| *Liparis campylostalix* Rchb.f. | 35.4375 | 129.2292 |
| *Liparis campylostalix* Rchb.f. | 35.4375 | 134.4375 |
| *Liparis campylostalix* Rchb.f. | 35.4375 | 137.7292 |
| *Liparis campylostalix* Rchb.f. | 35.4375 | 137.8542 |
| *Liparis campylostalix* Rchb.f. | 35.4375 | 138.0208 |
| *Liparis campylostalix* Rchb.f. | 35.4375 | 138.8125 |
| *Liparis campylostalix* Rchb.f. | 35.4375 | 138.9375 |
| *Liparis campylostalix* Rchb.f. | 35.4375 | 138.9792 |
| *Liparis campylostalix* Rchb.f. | 35.4375 | 139.0208 |
| *Liparis campylostalix* Rchb.f. | 35.4375 | 139.0625 |
| *Liparis campylostalix* Rchb.f. | 35.4375 | 139.1042 |
| *Liparis campylostalix* Rchb.f. | 35.4375 | 139.1458 |
| *Liparis campylostalix* Rchb.f. | 35.4375 | 139.1875 |
| *Liparis campylostalix* Rchb.f. | 35.4375 | 139.2292 |
| *Liparis campylostalix* Rchb.f. | 35.47917 | 126.8958 |
| *Liparis campylostalix* Rchb.f. | 35.47917 | 127.0625 |
| *Liparis campylostalix* Rchb.f. | 35.47917 | 127.4375 |
| *Liparis campylostalix* Rchb.f. | 35.47917 | 128.5625 |
| *Liparis campylostalix* Rchb.f. | 35.47917 | 134.6458 |
| *Liparis campylostalix* Rchb.f. | 35.47917 | 137.8125 |
| *Liparis campylostalix* Rchb.f. | 35.47917 | 137.8542 |
| *Liparis campylostalix* Rchb.f. | 35.47917 | 138.6875 |
| *Liparis campylostalix* Rchb.f. | 35.47917 | 139.0625 |
| *Liparis campylostalix* Rchb.f. | 35.47917 | 139.1458 |
| *Liparis campylostalix* Rchb.f. | 35.47917 | 139.1875 |
| *Liparis campylostalix* Rchb.f. | 35.52083 | 127.4792 |
| *Liparis campylostalix* Rchb.f. | 35.52083 | 127.9375 |
| *Liparis campylostalix* Rchb.f. | 35.52083 | 128.0208 |
| *Liparis campylostalix* Rchb.f. | 35.52083 | 137.8542 |
| *Liparis campylostalix* Rchb.f. | 35.52083 | 137.8958 |
| *Liparis campylostalix* Rchb.f. | 35.52083 | 137.9375 |
| *Liparis campylostalix* Rchb.f. | 35.52083 | 139.1458 |
| *Liparis campylostalix* Rchb.f. | 35.52083 | 139.1875 |
| *Liparis campylostalix* Rchb.f. | 35.52083 | 139.2708 |
| *Liparis campylostalix* Rchb.f. | 35.52083 | 139.5208 |
| *Liparis campylostalix* Rchb.f. | 35.5625 | 109.2292 |
| *Liparis campylostalix* Rchb.f. | 35.5625 | 127.2708 |
| *Liparis campylostalix* Rchb.f. | 35.5625 | 127.8542 |
| *Liparis campylostalix* Rchb.f. | 35.5625 | 128.8542 |
| *Liparis campylostalix* Rchb.f. | 35.5625 | 137.8542 |
| *Liparis campylostalix* Rchb.f. | 35.5625 | 137.9375 |
| *Liparis campylostalix* Rchb.f. | 35.5625 | 139.1458 |
| *Liparis campylostalix* Rchb.f. | 35.5625 | 139.2292 |
| *Liparis campylostalix* Rchb.f. | 35.5625 | 139.3542 |
| *Liparis campylostalix* Rchb.f. | 35.60417 | 127.4792 |
| *Liparis campylostalix* Rchb.f. | 35.60417 | 127.5208 |
| *Liparis campylostalix* Rchb.f. | 35.60417 | 127.6042 |
| *Liparis campylostalix* Rchb.f. | 35.60417 | 127.6458 |
| *Liparis campylostalix* Rchb.f. | 35.60417 | 127.9375 |
| *Liparis campylostalix* Rchb.f. | 35.60417 | 137.6875 |
| *Liparis campylostalix* Rchb.f. | 35.60417 | 137.8125 |
| *Liparis campylostalix* Rchb.f. | 35.60417 | 137.8542 |
| *Liparis campylostalix* Rchb.f. | 35.60417 | 138.0625 |
| *Liparis campylostalix* Rchb.f. | 35.60417 | 139.1458 |
| *Liparis campylostalix* Rchb.f. | 35.60417 | 139.1875 |
| *Liparis campylostalix* Rchb.f. | 35.60417 | 139.3125 |
| *Liparis campylostalix* Rchb.f. | 35.64583 | 138.0625 |
| *Liparis campylostalix* Rchb.f. | 35.64583 | 139.1458 |
| *Liparis campylostalix* Rchb.f. | 35.64583 | 139.1875 |
| *Liparis campylostalix* Rchb.f. | 35.6875 | 126.9792 |
| *Liparis campylostalix* Rchb.f. | 35.6875 | 127.4375 |
| *Liparis campylostalix* Rchb.f. | 35.6875 | 127.7708 |
| *Liparis campylostalix* Rchb.f. | 35.6875 | 137.6875 |
| *Liparis campylostalix* Rchb.f. | 35.6875 | 137.9375 |
| *Liparis campylostalix* Rchb.f. | 35.6875 | 139.6875 |
| *Liparis campylostalix* Rchb.f. | 35.72917 | 127.7708 |
| *Liparis campylostalix* Rchb.f. | 35.72917 | 128.7708 |
| *Liparis campylostalix* Rchb.f. | 35.72917 | 138.0625 |
| *Liparis campylostalix* Rchb.f. | 35.72917 | 138.1875 |
| *Liparis campylostalix* Rchb.f. | 35.72917 | 138.6042 |
| *Liparis campylostalix* Rchb.f. | 35.72917 | 138.6458 |
| *Liparis campylostalix* Rchb.f. | 35.77083 | 127.0625 |
| *Liparis campylostalix* Rchb.f. | 35.77083 | 128.7708 |
| *Liparis campylostalix* Rchb.f. | 35.8125 | 127.4375 |
| *Liparis campylostalix* Rchb.f. | 35.8125 | 137.9792 |
| *Liparis campylostalix* Rchb.f. | 35.8125 | 138.2292 |
| *Liparis campylostalix* Rchb.f. | 35.8125 | 138.6458 |
| *Liparis campylostalix* Rchb.f. | 35.8125 | 139.1458 |
| *Liparis campylostalix* Rchb.f. | 35.8125 | 139.1875 |
| *Liparis campylostalix* Rchb.f. | 35.8125 | 139.8958 |
| *Liparis campylostalix* Rchb.f. | 35.8125 | 140.0625 |
| *Liparis campylostalix* Rchb.f. | 35.85417 | 104.1875 |
| *Liparis campylostalix* Rchb.f. | 35.85417 | 127.5208 |
| *Liparis campylostalix* Rchb.f. | 35.85417 | 127.7708 |
| *Liparis campylostalix* Rchb.f. | 35.85417 | 127.8542 |
| *Liparis campylostalix* Rchb.f. | 35.85417 | 137.5625 |
| *Liparis campylostalix* Rchb.f. | 35.85417 | 137.6042 |
| *Liparis campylostalix* Rchb.f. | 35.85417 | 138.0625 |
| *Liparis campylostalix* Rchb.f. | 35.85417 | 138.2292 |
| *Liparis campylostalix* Rchb.f. | 35.89583 | 127.9792 |
| *Liparis campylostalix* Rchb.f. | 35.89583 | 137.5625 |
| *Liparis campylostalix* Rchb.f. | 35.89583 | 137.7708 |
| *Liparis campylostalix* Rchb.f. | 35.89583 | 137.9375 |
| *Liparis campylostalix* Rchb.f. | 35.89583 | 138.1875 |
| *Liparis campylostalix* Rchb.f. | 35.89583 | 138.2292 |
| *Liparis campylostalix* Rchb.f. | 35.89583 | 138.3125 |
| *Liparis campylostalix* Rchb.f. | 35.89583 | 138.4792 |
| *Liparis campylostalix* Rchb.f. | 35.9375 | 127.3958 |
| *Liparis campylostalix* Rchb.f. | 35.9375 | 137.8125 |
| *Liparis campylostalix* Rchb.f. | 35.9375 | 138.4792 |
| *Liparis campylostalix* Rchb.f. | 35.9375 | 138.6875 |
| *Liparis campylostalix* Rchb.f. | 35.9375 | 138.7292 |
| *Liparis campylostalix* Rchb.f. | 35.9375 | 139.1042 |
| *Liparis campylostalix* Rchb.f. | 35.97917 | 127.1042 |
| *Liparis campylostalix* Rchb.f. | 35.97917 | 138.1042 |
| *Liparis campylostalix* Rchb.f. | 35.97917 | 138.1458 |
| *Liparis campylostalix* Rchb.f. | 35.97917 | 138.1875 |
| *Liparis campylostalix* Rchb.f. | 35.97917 | 138.3125 |
| *Liparis campylostalix* Rchb.f. | 35.97917 | 138.3542 |
| *Liparis campylostalix* Rchb.f. | 35.97917 | 138.5208 |
| *Liparis campylostalix* Rchb.f. | 35.97917 | 138.6875 |
| *Liparis campylostalix* Rchb.f. | 36.02083 | 127.0208 |
| *Liparis campylostalix* Rchb.f. | 36.02083 | 127.0625 |
| *Liparis campylostalix* Rchb.f. | 36.02083 | 127.3542 |
| *Liparis campylostalix* Rchb.f. | 36.02083 | 127.6042 |
| *Liparis campylostalix* Rchb.f. | 36.02083 | 138.1042 |
| *Liparis campylostalix* Rchb.f. | 36.02083 | 138.1875 |
| *Liparis campylostalix* Rchb.f. | 36.02083 | 138.2292 |
| *Liparis campylostalix* Rchb.f. | 36.02083 | 140.0208 |
| *Liparis campylostalix* Rchb.f. | 36.0625 | 127.1458 |
| *Liparis campylostalix* Rchb.f. | 36.0625 | 127.3125 |
| *Liparis campylostalix* Rchb.f. | 36.0625 | 127.4375 |
| *Liparis campylostalix* Rchb.f. | 36.0625 | 127.6875 |
| *Liparis campylostalix* Rchb.f. | 36.0625 | 127.8125 |
| *Liparis campylostalix* Rchb.f. | 36.0625 | 128.4792 |
| *Liparis campylostalix* Rchb.f. | 36.0625 | 136.2292 |
| *Liparis campylostalix* Rchb.f. | 36.0625 | 137.8125 |
| *Liparis campylostalix* Rchb.f. | 36.0625 | 138.4375 |
| *Liparis campylostalix* Rchb.f. | 36.0625 | 138.5625 |
| *Liparis campylostalix* Rchb.f. | 36.0625 | 140.6042 |
| *Liparis campylostalix* Rchb.f. | 36.10417 | 127.6042 |
| *Liparis campylostalix* Rchb.f. | 36.10417 | 128.3125 |
| *Liparis campylostalix* Rchb.f. | 36.10417 | 136.7292 |
| *Liparis campylostalix* Rchb.f. | 36.10417 | 137.6042 |
| *Liparis campylostalix* Rchb.f. | 36.10417 | 137.6875 |
| *Liparis campylostalix* Rchb.f. | 36.10417 | 137.8125 |
| *Liparis campylostalix* Rchb.f. | 36.10417 | 137.9792 |
| *Liparis campylostalix* Rchb.f. | 36.10417 | 138.1875 |
| *Liparis campylostalix* Rchb.f. | 36.10417 | 138.2292 |
| *Liparis campylostalix* Rchb.f. | 36.10417 | 138.5625 |
| *Liparis campylostalix* Rchb.f. | 36.10417 | 138.6458 |
| *Liparis campylostalix* Rchb.f. | 36.10417 | 140.1042 |
| *Liparis campylostalix* Rchb.f. | 36.14583 | 126.8542 |
| *Liparis campylostalix* Rchb.f. | 36.14583 | 127.3542 |
| *Liparis campylostalix* Rchb.f. | 36.14583 | 128.5625 |
| *Liparis campylostalix* Rchb.f. | 36.14583 | 128.8542 |
| *Liparis campylostalix* Rchb.f. | 36.14583 | 128.9792 |
| *Liparis campylostalix* Rchb.f. | 36.14583 | 138.1875 |
| *Liparis campylostalix* Rchb.f. | 36.14583 | 138.2292 |
| *Liparis campylostalix* Rchb.f. | 36.1875 | 120.6042 |
| *Liparis campylostalix* Rchb.f. | 36.1875 | 120.6875 |
| *Liparis campylostalix* Rchb.f. | 36.1875 | 126.6875 |
| *Liparis campylostalix* Rchb.f. | 36.1875 | 127.6042 |
| *Liparis campylostalix* Rchb.f. | 36.1875 | 128.3125 |
| *Liparis campylostalix* Rchb.f. | 36.1875 | 137.8125 |
| *Liparis campylostalix* Rchb.f. | 36.1875 | 137.8542 |
| *Liparis campylostalix* Rchb.f. | 36.1875 | 138.1042 |
| *Liparis campylostalix* Rchb.f. | 36.1875 | 138.1875 |
| *Liparis campylostalix* Rchb.f. | 36.1875 | 138.2708 |
| *Liparis campylostalix* Rchb.f. | 36.1875 | 138.4375 |
| *Liparis campylostalix* Rchb.f. | 36.1875 | 138.4792 |
| *Liparis campylostalix* Rchb.f. | 36.1875 | 138.5625 |
| *Liparis campylostalix* Rchb.f. | 36.1875 | 138.6042 |
| *Liparis campylostalix* Rchb.f. | 36.1875 | 140.2708 |
| *Liparis campylostalix* Rchb.f. | 36.22917 | 120.6458 |
| *Liparis campylostalix* Rchb.f. | 36.22917 | 126.6042 |
| *Liparis campylostalix* Rchb.f. | 36.22917 | 127.1042 |
| *Liparis campylostalix* Rchb.f. | 36.22917 | 127.6042 |
| *Liparis campylostalix* Rchb.f. | 36.22917 | 127.9792 |
| *Liparis campylostalix* Rchb.f. | 36.22917 | 128.2292 |
| *Liparis campylostalix* Rchb.f. | 36.22917 | 128.4375 |
| *Liparis campylostalix* Rchb.f. | 36.22917 | 129.1458 |
| *Liparis campylostalix* Rchb.f. | 36.22917 | 137.8542 |
| *Liparis campylostalix* Rchb.f. | 36.22917 | 137.9792 |
| *Liparis campylostalix* Rchb.f. | 36.22917 | 138.0208 |
| *Liparis campylostalix* Rchb.f. | 36.22917 | 138.0625 |
| *Liparis campylostalix* Rchb.f. | 36.22917 | 138.1042 |
| *Liparis campylostalix* Rchb.f. | 36.22917 | 138.1875 |
| *Liparis campylostalix* Rchb.f. | 36.22917 | 138.2708 |
| *Liparis campylostalix* Rchb.f. | 36.22917 | 138.4792 |
| *Liparis campylostalix* Rchb.f. | 36.22917 | 140.1458 |
| *Liparis campylostalix* Rchb.f. | 36.22917 | 140.3958 |
| *Liparis campylostalix* Rchb.f. | 36.27083 | 126.6042 |
| *Liparis campylostalix* Rchb.f. | 36.27083 | 127.3125 |
| *Liparis campylostalix* Rchb.f. | 36.27083 | 128.2708 |
| *Liparis campylostalix* Rchb.f. | 36.27083 | 128.6042 |
| *Liparis campylostalix* Rchb.f. | 36.27083 | 128.7292 |
| *Liparis campylostalix* Rchb.f. | 36.27083 | 129.0625 |
| *Liparis campylostalix* Rchb.f. | 36.27083 | 136.8958 |
| *Liparis campylostalix* Rchb.f. | 36.27083 | 137.5625 |
| *Liparis campylostalix* Rchb.f. | 36.27083 | 138.0625 |
| *Liparis campylostalix* Rchb.f. | 36.27083 | 138.1042 |
| *Liparis campylostalix* Rchb.f. | 36.27083 | 138.1875 |
| *Liparis campylostalix* Rchb.f. | 36.27083 | 138.6042 |
| *Liparis campylostalix* Rchb.f. | 36.27083 | 139.8542 |
| *Liparis campylostalix* Rchb.f. | 36.27083 | 139.9792 |
| *Liparis campylostalix* Rchb.f. | 36.27083 | 140.3542 |
| *Liparis campylostalix* Rchb.f. | 36.3125 | 126.7708 |
| *Liparis campylostalix* Rchb.f. | 36.3125 | 127.0625 |
| *Liparis campylostalix* Rchb.f. | 36.3125 | 127.1875 |
| *Liparis campylostalix* Rchb.f. | 36.3125 | 128.0208 |
| *Liparis campylostalix* Rchb.f. | 36.3125 | 129.2708 |
| *Liparis campylostalix* Rchb.f. | 36.3125 | 137.0208 |
| *Liparis campylostalix* Rchb.f. | 36.3125 | 137.8125 |
| *Liparis campylostalix* Rchb.f. | 36.3125 | 137.8542 |
| *Liparis campylostalix* Rchb.f. | 36.3125 | 137.8958 |
| *Liparis campylostalix* Rchb.f. | 36.3125 | 138.2292 |
| *Liparis campylostalix* Rchb.f. | 36.3125 | 138.3125 |
| *Liparis campylostalix* Rchb.f. | 36.3125 | 140.1042 |
| *Liparis campylostalix* Rchb.f. | 36.3125 | 140.2292 |
| *Liparis campylostalix* Rchb.f. | 36.3125 | 140.3125 |
| *Liparis campylostalix* Rchb.f. | 36.35417 | 117.1042 |
| *Liparis campylostalix* Rchb.f. | 36.35417 | 126.6458 |
| *Liparis campylostalix* Rchb.f. | 36.35417 | 127.0625 |
| *Liparis campylostalix* Rchb.f. | 36.35417 | 128.1875 |
| *Liparis campylostalix* Rchb.f. | 36.35417 | 128.8125 |
| *Liparis campylostalix* Rchb.f. | 36.35417 | 128.9375 |
| *Liparis campylostalix* Rchb.f. | 36.35417 | 129.0625 |
| *Liparis campylostalix* Rchb.f. | 36.35417 | 129.1875 |
| *Liparis campylostalix* Rchb.f. | 36.35417 | 136.8125 |
| *Liparis campylostalix* Rchb.f. | 36.35417 | 137.8958 |
| *Liparis campylostalix* Rchb.f. | 36.35417 | 137.9375 |
| *Liparis campylostalix* Rchb.f. | 36.35417 | 137.9792 |
| *Liparis campylostalix* Rchb.f. | 36.35417 | 138.0625 |
| *Liparis campylostalix* Rchb.f. | 36.35417 | 138.1042 |
| *Liparis campylostalix* Rchb.f. | 36.35417 | 138.4792 |
| *Liparis campylostalix* Rchb.f. | 36.35417 | 138.5208 |
| *Liparis campylostalix* Rchb.f. | 36.35417 | 138.5625 |
| *Liparis campylostalix* Rchb.f. | 36.35417 | 138.6042 |
| *Liparis campylostalix* Rchb.f. | 36.35417 | 140.3125 |
| *Liparis campylostalix* Rchb.f. | 36.35417 | 140.4375 |
| *Liparis campylostalix* Rchb.f. | 36.39583 | 126.8542 |
| *Liparis campylostalix* Rchb.f. | 36.39583 | 126.8958 |
| *Liparis campylostalix* Rchb.f. | 36.39583 | 127.2292 |
| *Liparis campylostalix* Rchb.f. | 36.39583 | 127.2708 |
| *Liparis campylostalix* Rchb.f. | 36.39583 | 127.4375 |
| *Liparis campylostalix* Rchb.f. | 36.39583 | 137.1458 |
| *Liparis campylostalix* Rchb.f. | 36.39583 | 137.7292 |
| *Liparis campylostalix* Rchb.f. | 36.39583 | 137.9792 |
| *Liparis campylostalix* Rchb.f. | 36.39583 | 138.0625 |
| *Liparis campylostalix* Rchb.f. | 36.39583 | 138.1042 |
| *Liparis campylostalix* Rchb.f. | 36.39583 | 138.1458 |
| *Liparis campylostalix* Rchb.f. | 36.39583 | 138.3958 |
| *Liparis campylostalix* Rchb.f. | 36.39583 | 138.5625 |
| *Liparis campylostalix* Rchb.f. | 36.39583 | 140.2292 |
| *Liparis campylostalix* Rchb.f. | 36.39583 | 140.3958 |
| *Liparis campylostalix* Rchb.f. | 36.39583 | 140.4792 |
| *Liparis campylostalix* Rchb.f. | 36.4375 | 126.3542 |
| *Liparis campylostalix* Rchb.f. | 36.4375 | 126.4375 |
| *Liparis campylostalix* Rchb.f. | 36.4375 | 126.6042 |
| *Liparis campylostalix* Rchb.f. | 36.4375 | 126.8125 |
| *Liparis campylostalix* Rchb.f. | 36.4375 | 126.8542 |
| *Liparis campylostalix* Rchb.f. | 36.4375 | 126.8958 |
| *Liparis campylostalix* Rchb.f. | 36.4375 | 128.1042 |
| *Liparis campylostalix* Rchb.f. | 36.4375 | 128.1458 |
| *Liparis campylostalix* Rchb.f. | 36.4375 | 136.8958 |
| *Liparis campylostalix* Rchb.f. | 36.4375 | 137.8125 |
| *Liparis campylostalix* Rchb.f. | 36.4375 | 137.8542 |
| *Liparis campylostalix* Rchb.f. | 36.4375 | 137.9375 |
| *Liparis campylostalix* Rchb.f. | 36.4375 | 137.9792 |
| *Liparis campylostalix* Rchb.f. | 36.4375 | 138.0625 |
| *Liparis campylostalix* Rchb.f. | 36.4375 | 138.2708 |
| *Liparis campylostalix* Rchb.f. | 36.4375 | 138.4375 |
| *Liparis campylostalix* Rchb.f. | 36.4375 | 140.0208 |
| *Liparis campylostalix* Rchb.f. | 36.47917 | 126.1458 |
| *Liparis campylostalix* Rchb.f. | 36.47917 | 126.6875 |
| *Liparis campylostalix* Rchb.f. | 36.47917 | 127.0208 |
| *Liparis campylostalix* Rchb.f. | 36.47917 | 127.9792 |
| *Liparis campylostalix* Rchb.f. | 36.47917 | 128.3542 |
| *Liparis campylostalix* Rchb.f. | 36.47917 | 128.7708 |
| *Liparis campylostalix* Rchb.f. | 36.47917 | 128.8958 |
| *Liparis campylostalix* Rchb.f. | 36.47917 | 136.9375 |
| *Liparis campylostalix* Rchb.f. | 36.47917 | 137.0208 |
| *Liparis campylostalix* Rchb.f. | 36.47917 | 137.9375 |
| *Liparis campylostalix* Rchb.f. | 36.47917 | 138.0625 |
| *Liparis campylostalix* Rchb.f. | 36.47917 | 138.1042 |
| *Liparis campylostalix* Rchb.f. | 36.47917 | 138.1458 |
| *Liparis campylostalix* Rchb.f. | 36.47917 | 138.3542 |
| *Liparis campylostalix* Rchb.f. | 36.47917 | 138.8958 |
| *Liparis campylostalix* Rchb.f. | 36.47917 | 138.9792 |
| *Liparis campylostalix* Rchb.f. | 36.47917 | 139.2708 |
| *Liparis campylostalix* Rchb.f. | 36.47917 | 140.3542 |
| *Liparis campylostalix* Rchb.f. | 36.47917 | 140.3958 |
| *Liparis campylostalix* Rchb.f. | 36.52083 | 103.8958 |
| *Liparis campylostalix* Rchb.f. | 36.52083 | 126.3542 |
| *Liparis campylostalix* Rchb.f. | 36.52083 | 128.0208 |
| *Liparis campylostalix* Rchb.f. | 36.52083 | 137.9792 |
| *Liparis campylostalix* Rchb.f. | 36.52083 | 138.0208 |
| *Liparis campylostalix* Rchb.f. | 36.52083 | 138.1042 |
| *Liparis campylostalix* Rchb.f. | 36.52083 | 138.2292 |
| *Liparis campylostalix* Rchb.f. | 36.52083 | 138.3542 |
| *Liparis campylostalix* Rchb.f. | 36.52083 | 140.5208 |
| *Liparis campylostalix* Rchb.f. | 36.52083 | 140.5625 |
| *Liparis campylostalix* Rchb.f. | 36.5625 | 127.0625 |
| *Liparis campylostalix* Rchb.f. | 36.5625 | 127.1458 |
| *Liparis campylostalix* Rchb.f. | 36.5625 | 127.1875 |
| *Liparis campylostalix* Rchb.f. | 36.5625 | 127.6875 |
| *Liparis campylostalix* Rchb.f. | 36.5625 | 127.8542 |
| *Liparis campylostalix* Rchb.f. | 36.5625 | 129.0625 |
| *Liparis campylostalix* Rchb.f. | 36.5625 | 129.2708 |
| *Liparis campylostalix* Rchb.f. | 36.5625 | 136.9375 |
| *Liparis campylostalix* Rchb.f. | 36.5625 | 137.2292 |
| *Liparis campylostalix* Rchb.f. | 36.5625 | 137.3542 |
| *Liparis campylostalix* Rchb.f. | 36.5625 | 137.3958 |
| *Liparis campylostalix* Rchb.f. | 36.5625 | 137.4375 |
| *Liparis campylostalix* Rchb.f. | 36.5625 | 137.5208 |
| *Liparis campylostalix* Rchb.f. | 36.5625 | 137.8542 |
| *Liparis campylostalix* Rchb.f. | 36.5625 | 138.0625 |
| *Liparis campylostalix* Rchb.f. | 36.5625 | 138.1042 |
| *Liparis campylostalix* Rchb.f. | 36.5625 | 138.3125 |
| *Liparis campylostalix* Rchb.f. | 36.5625 | 138.4792 |
| *Liparis campylostalix* Rchb.f. | 36.5625 | 139.0208 |
| *Liparis campylostalix* Rchb.f. | 36.5625 | 139.1875 |
| *Liparis campylostalix* Rchb.f. | 36.5625 | 140.3958 |
| *Liparis campylostalix* Rchb.f. | 36.5625 | 140.5208 |
| *Liparis campylostalix* Rchb.f. | 36.5625 | 140.5625 |
| *Liparis campylostalix* Rchb.f. | 36.60417 | 127.1458 |
| *Liparis campylostalix* Rchb.f. | 36.60417 | 128.1458 |
| *Liparis campylostalix* Rchb.f. | 36.60417 | 129.2292 |
| *Liparis campylostalix* Rchb.f. | 36.60417 | 137.0625 |
| *Liparis campylostalix* Rchb.f. | 36.60417 | 137.8542 |
| *Liparis campylostalix* Rchb.f. | 36.60417 | 139.3958 |
| *Liparis campylostalix* Rchb.f. | 36.60417 | 140.6458 |
| *Liparis campylostalix* Rchb.f. | 36.64583 | 137.3958 |
| *Liparis campylostalix* Rchb.f. | 36.64583 | 138.1042 |
| *Liparis campylostalix* Rchb.f. | 36.64583 | 138.1875 |
| *Liparis campylostalix* Rchb.f. | 36.64583 | 139.1042 |
| *Liparis campylostalix* Rchb.f. | 36.6875 | 127.2292 |
| *Liparis campylostalix* Rchb.f. | 36.6875 | 128.0208 |
| *Liparis campylostalix* Rchb.f. | 36.6875 | 128.3542 |
| *Liparis campylostalix* Rchb.f. | 36.6875 | 136.8125 |
| *Liparis campylostalix* Rchb.f. | 36.6875 | 137.0625 |
| *Liparis campylostalix* Rchb.f. | 36.6875 | 137.1458 |
| *Liparis campylostalix* Rchb.f. | 36.6875 | 137.5208 |
| *Liparis campylostalix* Rchb.f. | 36.6875 | 138.4792 |
| *Liparis campylostalix* Rchb.f. | 36.6875 | 138.5208 |
| *Liparis campylostalix* Rchb.f. | 36.6875 | 139.1042 |
| *Liparis campylostalix* Rchb.f. | 36.72917 | 127.6458 |
| *Liparis campylostalix* Rchb.f. | 36.72917 | 128.1875 |
| *Liparis campylostalix* Rchb.f. | 36.72917 | 128.3125 |
| *Liparis campylostalix* Rchb.f. | 36.72917 | 137.0625 |
| *Liparis campylostalix* Rchb.f. | 36.72917 | 137.4375 |
| *Liparis campylostalix* Rchb.f. | 36.72917 | 137.4792 |
| *Liparis campylostalix* Rchb.f. | 36.72917 | 137.5625 |
| *Liparis campylostalix* Rchb.f. | 36.72917 | 137.9375 |
| *Liparis campylostalix* Rchb.f. | 36.72917 | 138.1042 |
| *Liparis campylostalix* Rchb.f. | 36.72917 | 138.1458 |
| *Liparis campylostalix* Rchb.f. | 36.72917 | 138.3125 |
| *Liparis campylostalix* Rchb.f. | 36.72917 | 138.4375 |
| *Liparis campylostalix* Rchb.f. | 36.72917 | 138.5625 |
| *Liparis campylostalix* Rchb.f. | 36.72917 | 138.6458 |
| *Liparis campylostalix* Rchb.f. | 36.72917 | 140.4792 |
| *Liparis campylostalix* Rchb.f. | 36.72917 | 140.7292 |
| *Liparis campylostalix* Rchb.f. | 36.77083 | 126.1875 |
| *Liparis campylostalix* Rchb.f. | 36.77083 | 126.3125 |
| *Liparis campylostalix* Rchb.f. | 36.77083 | 126.6042 |
| *Liparis campylostalix* Rchb.f. | 36.77083 | 127.0625 |
| *Liparis campylostalix* Rchb.f. | 36.77083 | 128.9375 |
| *Liparis campylostalix* Rchb.f. | 36.77083 | 137.3958 |
| *Liparis campylostalix* Rchb.f. | 36.77083 | 137.4375 |
| *Liparis campylostalix* Rchb.f. | 36.77083 | 137.5625 |
| *Liparis campylostalix* Rchb.f. | 36.77083 | 137.8958 |
| *Liparis campylostalix* Rchb.f. | 36.77083 | 138.0625 |
| *Liparis campylostalix* Rchb.f. | 36.77083 | 138.1042 |
| *Liparis campylostalix* Rchb.f. | 36.77083 | 138.6458 |
| *Liparis campylostalix* Rchb.f. | 36.77083 | 138.6875 |
| *Liparis campylostalix* Rchb.f. | 36.77083 | 139.6042 |
| *Liparis campylostalix* Rchb.f. | 36.77083 | 140.3542 |
| *Liparis campylostalix* Rchb.f. | 36.8125 | 101.9375 |
| *Liparis campylostalix* Rchb.f. | 36.8125 | 120.4792 |
| *Liparis campylostalix* Rchb.f. | 36.8125 | 126.1458 |
| *Liparis campylostalix* Rchb.f. | 36.8125 | 126.3958 |
| *Liparis campylostalix* Rchb.f. | 36.8125 | 127.2292 |
| *Liparis campylostalix* Rchb.f. | 36.8125 | 127.3125 |
| *Liparis campylostalix* Rchb.f. | 36.8125 | 127.6875 |
| *Liparis campylostalix* Rchb.f. | 36.8125 | 128.1042 |
| *Liparis campylostalix* Rchb.f. | 36.8125 | 128.3542 |
| *Liparis campylostalix* Rchb.f. | 36.8125 | 128.8125 |
| *Liparis campylostalix* Rchb.f. | 36.8125 | 128.8542 |
| *Liparis campylostalix* Rchb.f. | 36.8125 | 129.0208 |
| *Liparis campylostalix* Rchb.f. | 36.8125 | 129.1042 |
| *Liparis campylostalix* Rchb.f. | 36.8125 | 137.0208 |
| *Liparis campylostalix* Rchb.f. | 36.8125 | 138.1875 |
| *Liparis campylostalix* Rchb.f. | 36.8125 | 138.5625 |
| *Liparis campylostalix* Rchb.f. | 36.8125 | 139.3958 |
| *Liparis campylostalix* Rchb.f. | 36.8125 | 140.7292 |
| *Liparis campylostalix* Rchb.f. | 36.85417 | 102.1458 |
| *Liparis campylostalix* Rchb.f. | 36.85417 | 126.1875 |
| *Liparis campylostalix* Rchb.f. | 36.85417 | 127.2292 |
| *Liparis campylostalix* Rchb.f. | 36.85417 | 127.3542 |
| *Liparis campylostalix* Rchb.f. | 36.85417 | 127.3958 |
| *Liparis campylostalix* Rchb.f. | 36.85417 | 128.0208 |
| *Liparis campylostalix* Rchb.f. | 36.85417 | 128.1042 |
| *Liparis campylostalix* Rchb.f. | 36.85417 | 128.3125 |
| *Liparis campylostalix* Rchb.f. | 36.85417 | 128.3542 |
| *Liparis campylostalix* Rchb.f. | 36.85417 | 137.5625 |
| *Liparis campylostalix* Rchb.f. | 36.85417 | 138.1875 |
| *Liparis campylostalix* Rchb.f. | 36.85417 | 138.6458 |
| *Liparis campylostalix* Rchb.f. | 36.85417 | 138.6875 |
| *Liparis campylostalix* Rchb.f. | 36.85417 | 140.7708 |
| *Liparis campylostalix* Rchb.f. | 36.89583 | 126.1875 |
| *Liparis campylostalix* Rchb.f. | 36.89583 | 126.9792 |
| *Liparis campylostalix* Rchb.f. | 36.89583 | 128.3958 |
| *Liparis campylostalix* Rchb.f. | 36.89583 | 137.9375 |
| *Liparis campylostalix* Rchb.f. | 36.89583 | 137.9792 |
| *Liparis campylostalix* Rchb.f. | 36.89583 | 138.3542 |
| *Liparis campylostalix* Rchb.f. | 36.89583 | 138.4375 |
| *Liparis campylostalix* Rchb.f. | 36.89583 | 138.7292 |
| *Liparis campylostalix* Rchb.f. | 36.89583 | 138.7708 |
| *Liparis campylostalix* Rchb.f. | 36.89583 | 139.5625 |
| *Liparis campylostalix* Rchb.f. | 36.9375 | 127.2708 |
| *Liparis campylostalix* Rchb.f. | 36.9375 | 127.3125 |
| *Liparis campylostalix* Rchb.f. | 36.9375 | 128.5625 |
| *Liparis campylostalix* Rchb.f. | 36.9375 | 128.6875 |
| *Liparis campylostalix* Rchb.f. | 36.9375 | 128.8125 |
| *Liparis campylostalix* Rchb.f. | 36.9375 | 128.8958 |
| *Liparis campylostalix* Rchb.f. | 36.9375 | 129.2708 |
| *Liparis campylostalix* Rchb.f. | 36.9375 | 137.6042 |
| *Liparis campylostalix* Rchb.f. | 36.9375 | 137.6458 |
| *Liparis campylostalix* Rchb.f. | 36.9375 | 138.4375 |
| *Liparis campylostalix* Rchb.f. | 36.9375 | 139.9375 |
| *Liparis campylostalix* Rchb.f. | 36.97917 | 128.2292 |
| *Liparis campylostalix* Rchb.f. | 36.97917 | 128.8125 |
| *Liparis campylostalix* Rchb.f. | 36.97917 | 128.9375 |
| *Liparis campylostalix* Rchb.f. | 36.97917 | 129.0625 |
| *Liparis campylostalix* Rchb.f. | 36.97917 | 129.1458 |
| *Liparis campylostalix* Rchb.f. | 36.97917 | 129.3125 |
| *Liparis campylostalix* Rchb.f. | 36.97917 | 139.9792 |
| *Liparis campylostalix* Rchb.f. | 37.02083 | 127.3958 |
| *Liparis campylostalix* Rchb.f. | 37.02083 | 127.6458 |
| *Liparis campylostalix* Rchb.f. | 37.02083 | 128.8125 |
| *Liparis campylostalix* Rchb.f. | 37.02083 | 128.8958 |
| *Liparis campylostalix* Rchb.f. | 37.02083 | 129.2292 |
| *Liparis campylostalix* Rchb.f. | 37.02083 | 138.7292 |
| *Liparis campylostalix* Rchb.f. | 37.02083 | 140.3958 |
| *Liparis campylostalix* Rchb.f. | 37.0625 | 126.9375 |
| *Liparis campylostalix* Rchb.f. | 37.0625 | 127.6875 |
| *Liparis campylostalix* Rchb.f. | 37.0625 | 127.7708 |
| *Liparis campylostalix* Rchb.f. | 37.0625 | 128.5625 |
| *Liparis campylostalix* Rchb.f. | 37.0625 | 129.1458 |
| *Liparis campylostalix* Rchb.f. | 37.0625 | 138.1875 |
| *Liparis campylostalix* Rchb.f. | 37.10417 | 128.4375 |
| *Liparis campylostalix* Rchb.f. | 37.10417 | 128.6875 |
| *Liparis campylostalix* Rchb.f. | 37.10417 | 128.8958 |
| *Liparis campylostalix* Rchb.f. | 37.10417 | 129.0625 |
| *Liparis campylostalix* Rchb.f. | 37.10417 | 138.2292 |
| *Liparis campylostalix* Rchb.f. | 37.10417 | 140.2292 |
| *Liparis campylostalix* Rchb.f. | 37.14583 | 127.4375 |
| *Liparis campylostalix* Rchb.f. | 37.14583 | 127.5625 |
| *Liparis campylostalix* Rchb.f. | 37.14583 | 127.6875 |
| *Liparis campylostalix* Rchb.f. | 37.14583 | 128.8958 |
| *Liparis campylostalix* Rchb.f. | 37.1875 | 126.9375 |
| *Liparis campylostalix* Rchb.f. | 37.1875 | 127.2708 |
| *Liparis campylostalix* Rchb.f. | 37.1875 | 127.8542 |
| *Liparis campylostalix* Rchb.f. | 37.1875 | 128.3542 |
| *Liparis campylostalix* Rchb.f. | 37.1875 | 128.5625 |
| *Liparis campylostalix* Rchb.f. | 37.1875 | 128.6042 |
| *Liparis campylostalix* Rchb.f. | 37.1875 | 128.7708 |
| *Liparis campylostalix* Rchb.f. | 37.1875 | 129.0208 |
| *Liparis campylostalix* Rchb.f. | 37.1875 | 129.1875 |
| *Liparis campylostalix* Rchb.f. | 37.22917 | 127.1875 |
| *Liparis campylostalix* Rchb.f. | 37.22917 | 128.1458 |
| *Liparis campylostalix* Rchb.f. | 37.22917 | 129.1042 |
| *Liparis campylostalix* Rchb.f. | 37.22917 | 139.6042 |
| *Liparis campylostalix* Rchb.f. | 37.27083 | 114.5208 |
| *Liparis campylostalix* Rchb.f. | 37.27083 | 126.9375 |
| *Liparis campylostalix* Rchb.f. | 37.27083 | 127.0208 |
| *Liparis campylostalix* Rchb.f. | 37.27083 | 127.6042 |
| *Liparis campylostalix* Rchb.f. | 37.27083 | 127.8542 |
| *Liparis campylostalix* Rchb.f. | 37.27083 | 128.5208 |
| *Liparis campylostalix* Rchb.f. | 37.27083 | 128.5625 |
| *Liparis campylostalix* Rchb.f. | 37.27083 | 128.6042 |
| *Liparis campylostalix* Rchb.f. | 37.27083 | 128.8542 |
| *Liparis campylostalix* Rchb.f. | 37.27083 | 128.9792 |
| *Liparis campylostalix* Rchb.f. | 37.27083 | 138.9792 |
| *Liparis campylostalix* Rchb.f. | 37.3125 | 114.5208 |
| *Liparis campylostalix* Rchb.f. | 37.3125 | 127.2708 |
| *Liparis campylostalix* Rchb.f. | 37.3125 | 128.3125 |
| *Liparis campylostalix* Rchb.f. | 37.3125 | 128.6458 |
| *Liparis campylostalix* Rchb.f. | 37.3125 | 129.1875 |
| *Liparis campylostalix* Rchb.f. | 37.35417 | 127.0208 |
| *Liparis campylostalix* Rchb.f. | 37.35417 | 127.0625 |
| *Liparis campylostalix* Rchb.f. | 37.35417 | 127.1875 |
| *Liparis campylostalix* Rchb.f. | 37.35417 | 127.2708 |
| *Liparis campylostalix* Rchb.f. | 37.35417 | 128.0625 |
| *Liparis campylostalix* Rchb.f. | 37.35417 | 128.1042 |
| *Liparis campylostalix* Rchb.f. | 37.35417 | 140.6875 |
| *Liparis campylostalix* Rchb.f. | 37.39583 | 127.0208 |
| *Liparis campylostalix* Rchb.f. | 37.39583 | 127.0625 |
| *Liparis campylostalix* Rchb.f. | 37.39583 | 127.1458 |
| *Liparis campylostalix* Rchb.f. | 37.39583 | 128.2708 |
| *Liparis campylostalix* Rchb.f. | 37.39583 | 128.3125 |
| *Liparis campylostalix* Rchb.f. | 37.39583 | 128.5208 |
| *Liparis campylostalix* Rchb.f. | 37.39583 | 128.8125 |
| *Liparis campylostalix* Rchb.f. | 37.39583 | 128.8542 |
| *Liparis campylostalix* Rchb.f. | 37.39583 | 128.8958 |
| *Liparis campylostalix* Rchb.f. | 37.39583 | 140.3542 |
| *Liparis campylostalix* Rchb.f. | 37.39583 | 140.9792 |
| *Liparis campylostalix* Rchb.f. | 37.4375 | 127.0208 |
| *Liparis campylostalix* Rchb.f. | 37.4375 | 127.5625 |
| *Liparis campylostalix* Rchb.f. | 37.4375 | 127.8542 |
| *Liparis campylostalix* Rchb.f. | 37.4375 | 128.1458 |
| *Liparis campylostalix* Rchb.f. | 37.4375 | 128.7292 |
| *Liparis campylostalix* Rchb.f. | 37.4375 | 128.8958 |
| *Liparis campylostalix* Rchb.f. | 37.47917 | 126.5208 |
| *Liparis campylostalix* Rchb.f. | 37.47917 | 127.7292 |
| *Liparis campylostalix* Rchb.f. | 37.47917 | 128.6458 |
| *Liparis campylostalix* Rchb.f. | 37.47917 | 128.6875 |
| *Liparis campylostalix* Rchb.f. | 37.47917 | 128.8958 |
| *Liparis campylostalix* Rchb.f. | 37.47917 | 130.9792 |
| *Liparis campylostalix* Rchb.f. | 37.47917 | 140.1042 |
| *Liparis campylostalix* Rchb.f. | 37.47917 | 140.1458 |
| *Liparis campylostalix* Rchb.f. | 37.52083 | 128.5208 |
| *Liparis campylostalix* Rchb.f. | 37.52083 | 129.0208 |
| *Liparis campylostalix* Rchb.f. | 37.52083 | 139.6875 |
| *Liparis campylostalix* Rchb.f. | 37.5625 | 127.2708 |
| *Liparis campylostalix* Rchb.f. | 37.5625 | 127.5625 |
| *Liparis campylostalix* Rchb.f. | 37.5625 | 127.8542 |
| *Liparis campylostalix* Rchb.f. | 37.5625 | 128.3958 |
| *Liparis campylostalix* Rchb.f. | 37.5625 | 128.5208 |
| *Liparis campylostalix* Rchb.f. | 37.5625 | 128.6875 |
| *Liparis campylostalix* Rchb.f. | 37.5625 | 128.7708 |
| *Liparis campylostalix* Rchb.f. | 37.60417 | 127.4792 |
| *Liparis campylostalix* Rchb.f. | 37.60417 | 128.0625 |
| *Liparis campylostalix* Rchb.f. | 37.60417 | 128.2708 |
| *Liparis campylostalix* Rchb.f. | 37.60417 | 128.3125 |
| *Liparis campylostalix* Rchb.f. | 37.60417 | 128.6458 |
| *Liparis campylostalix* Rchb.f. | 37.60417 | 128.6875 |
| *Liparis campylostalix* Rchb.f. | 37.60417 | 140.0625 |
| *Liparis campylostalix* Rchb.f. | 37.64583 | 125.6875 |
| *Liparis campylostalix* Rchb.f. | 37.64583 | 127.1042 |
| *Liparis campylostalix* Rchb.f. | 37.64583 | 128.1458 |
| *Liparis campylostalix* Rchb.f. | 37.64583 | 128.1875 |
| *Liparis campylostalix* Rchb.f. | 37.64583 | 128.3125 |
| *Liparis campylostalix* Rchb.f. | 37.64583 | 128.5625 |
| *Liparis campylostalix* Rchb.f. | 37.64583 | 128.6042 |
| *Liparis campylostalix* Rchb.f. | 37.64583 | 129.0208 |
| *Liparis campylostalix* Rchb.f. | 37.64583 | 140.1042 |
| *Liparis campylostalix* Rchb.f. | 37.64583 | 140.3958 |
| *Liparis campylostalix* Rchb.f. | 37.6875 | 114.3958 |
| *Liparis campylostalix* Rchb.f. | 37.6875 | 127.2708 |
| *Liparis campylostalix* Rchb.f. | 37.6875 | 127.4375 |
| *Liparis campylostalix* Rchb.f. | 37.6875 | 128.8958 |
| *Liparis campylostalix* Rchb.f. | 37.6875 | 140.0625 |
| *Liparis campylostalix* Rchb.f. | 37.6875 | 140.1042 |
| *Liparis campylostalix* Rchb.f. | 37.6875 | 140.4375 |
| *Liparis campylostalix* Rchb.f. | 37.6875 | 140.4792 |
| *Liparis campylostalix* Rchb.f. | 37.72917 | 127.2292 |
| *Liparis campylostalix* Rchb.f. | 37.72917 | 128.1042 |
| *Liparis campylostalix* Rchb.f. | 37.72917 | 128.6042 |
| *Liparis campylostalix* Rchb.f. | 37.72917 | 139.4375 |
| *Liparis campylostalix* Rchb.f. | 37.72917 | 139.8542 |
| *Liparis campylostalix* Rchb.f. | 37.72917 | 139.9375 |
| *Liparis campylostalix* Rchb.f. | 37.72917 | 140.3542 |
| *Liparis campylostalix* Rchb.f. | 37.77083 | 126.2708 |
| *Liparis campylostalix* Rchb.f. | 37.77083 | 127.1875 |
| *Liparis campylostalix* Rchb.f. | 37.77083 | 127.8542 |
| *Liparis campylostalix* Rchb.f. | 37.77083 | 128.3958 |
| *Liparis campylostalix* Rchb.f. | 37.77083 | 140.6875 |
| *Liparis campylostalix* Rchb.f. | 37.77083 | 140.8542 |
| *Liparis campylostalix* Rchb.f. | 37.8125 | 126.4375 |
| *Liparis campylostalix* Rchb.f. | 37.8125 | 127.6042 |
| *Liparis campylostalix* Rchb.f. | 37.8125 | 128.3125 |
| *Liparis campylostalix* Rchb.f. | 37.8125 | 128.7708 |
| *Liparis campylostalix* Rchb.f. | 37.8125 | 140.9375 |
| *Liparis campylostalix* Rchb.f. | 37.8125 | 140.9792 |
| *Liparis campylostalix* Rchb.f. | 37.85417 | 124.7292 |
| *Liparis campylostalix* Rchb.f. | 37.85417 | 128.1458 |
| *Liparis campylostalix* Rchb.f. | 37.89583 | 112.0208 |
| *Liparis campylostalix* Rchb.f. | 37.89583 | 128.1875 |
| *Liparis campylostalix* Rchb.f. | 37.89583 | 128.3125 |
| *Liparis campylostalix* Rchb.f. | 37.9375 | 126.9792 |
| *Liparis campylostalix* Rchb.f. | 37.97917 | 128.0625 |
| *Liparis campylostalix* Rchb.f. | 37.97917 | 139.9375 |
| *Liparis campylostalix* Rchb.f. | 38.0625 | 127.3958 |
| *Liparis campylostalix* Rchb.f. | 38.0625 | 127.7292 |
| *Liparis campylostalix* Rchb.f. | 38.0625 | 127.8958 |
| *Liparis campylostalix* Rchb.f. | 38.0625 | 128.0208 |
| *Liparis campylostalix* Rchb.f. | 38.0625 | 128.2708 |
| *Liparis campylostalix* Rchb.f. | 38.0625 | 128.4375 |
| *Liparis campylostalix* Rchb.f. | 38.0625 | 128.4792 |
| *Liparis campylostalix* Rchb.f. | 38.10417 | 127.0625 |
| *Liparis campylostalix* Rchb.f. | 38.10417 | 128.1458 |
| *Liparis campylostalix* Rchb.f. | 38.14583 | 127.1875 |
| *Liparis campylostalix* Rchb.f. | 38.14583 | 127.3125 |
| *Liparis campylostalix* Rchb.f. | 38.14583 | 140.3958 |
| *Liparis campylostalix* Rchb.f. | 38.14583 | 140.4375 |
| *Liparis campylostalix* Rchb.f. | 38.1875 | 127.3958 |
| *Liparis campylostalix* Rchb.f. | 38.1875 | 127.4792 |
| *Liparis campylostalix* Rchb.f. | 38.1875 | 127.5208 |
| *Liparis campylostalix* Rchb.f. | 38.1875 | 128.0208 |
| *Liparis campylostalix* Rchb.f. | 38.1875 | 128.1042 |
| *Liparis campylostalix* Rchb.f. | 38.1875 | 140.1458 |
| *Liparis campylostalix* Rchb.f. | 38.1875 | 140.2708 |
| *Liparis campylostalix* Rchb.f. | 38.22917 | 114.1875 |
| *Liparis campylostalix* Rchb.f. | 38.22917 | 127.5208 |
| *Liparis campylostalix* Rchb.f. | 38.22917 | 128.1458 |
| *Liparis campylostalix* Rchb.f. | 38.22917 | 128.1875 |
| *Liparis campylostalix* Rchb.f. | 38.22917 | 139.9792 |
| *Liparis campylostalix* Rchb.f. | 38.22917 | 140.1875 |
| *Liparis campylostalix* Rchb.f. | 38.22917 | 140.3542 |
| *Liparis campylostalix* Rchb.f. | 38.22917 | 140.8125 |
| *Liparis campylostalix* Rchb.f. | 38.27083 | 140.3958 |
| *Liparis campylostalix* Rchb.f. | 38.3125 | 128.2292 |
| *Liparis campylostalix* Rchb.f. | 38.3125 | 140.4375 |
| *Liparis campylostalix* Rchb.f. | 38.3125 | 140.4792 |
| *Liparis campylostalix* Rchb.f. | 38.3125 | 140.9375 |
| *Liparis campylostalix* Rchb.f. | 38.35417 | 127.2708 |
| *Liparis campylostalix* Rchb.f. | 38.35417 | 139.9792 |
| *Liparis campylostalix* Rchb.f. | 38.39583 | 141.6875 |
| *Liparis campylostalix* Rchb.f. | 38.4375 | 140.6042 |
| *Liparis campylostalix* Rchb.f. | 38.47917 | 113.8958 |
| *Liparis campylostalix* Rchb.f. | 38.47917 | 128.3958 |
| *Liparis campylostalix* Rchb.f. | 38.47917 | 128.4375 |
| *Liparis campylostalix* Rchb.f. | 38.47917 | 140.3542 |
| *Liparis campylostalix* Rchb.f. | 38.64583 | 128.0625 |
| *Liparis campylostalix* Rchb.f. | 38.77083 | 140.3958 |
| *Liparis campylostalix* Rchb.f. | 38.77083 | 141.0208 |
| *Liparis campylostalix* Rchb.f. | 38.8125 | 140.2292 |
| *Liparis campylostalix* Rchb.f. | 38.8125 | 140.8125 |
| *Liparis campylostalix* Rchb.f. | 38.9375 | 140.5208 |
| *Liparis campylostalix* Rchb.f. | 39.10417 | 139.8542 |
| *Liparis campylostalix* Rchb.f. | 39.10417 | 140.0625 |
| *Liparis campylostalix* Rchb.f. | 39.10417 | 140.7292 |
| *Liparis campylostalix* Rchb.f. | 39.14583 | 140.4792 |
| *Liparis campylostalix* Rchb.f. | 39.14583 | 141.5625 |
| *Liparis campylostalix* Rchb.f. | 39.1875 | 140.4792 |
| *Liparis campylostalix* Rchb.f. | 39.1875 | 140.6458 |
| *Liparis campylostalix* Rchb.f. | 39.1875 | 140.8958 |
| *Liparis campylostalix* Rchb.f. | 39.27083 | 139.9792 |
| *Liparis campylostalix* Rchb.f. | 39.27083 | 141.1042 |
| *Liparis campylostalix* Rchb.f. | 39.27083 | 141.3542 |
| *Liparis campylostalix* Rchb.f. | 39.27083 | 141.7708 |
| *Liparis campylostalix* Rchb.f. | 39.39583 | 140.0625 |
| *Liparis campylostalix* Rchb.f. | 39.4375 | 140.4792 |
| *Liparis campylostalix* Rchb.f. | 39.4375 | 140.6875 |
| *Liparis campylostalix* Rchb.f. | 39.47917 | 140.5625 |
| *Liparis campylostalix* Rchb.f. | 39.47917 | 140.6875 |
| *Liparis campylostalix* Rchb.f. | 39.52083 | 140.6458 |
| *Liparis campylostalix* Rchb.f. | 39.52083 | 140.6875 |
| *Liparis campylostalix* Rchb.f. | 39.60417 | 140.6458 |
| *Liparis campylostalix* Rchb.f. | 39.60417 | 140.7708 |
| *Liparis campylostalix* Rchb.f. | 39.60417 | 141.0208 |
| *Liparis campylostalix* Rchb.f. | 39.64583 | 141.9375 |
| *Liparis campylostalix* Rchb.f. | 39.6875 | 122.9792 |
| *Liparis campylostalix* Rchb.f. | 39.6875 | 140.6875 |
| *Liparis campylostalix* Rchb.f. | 39.6875 | 140.7292 |
| *Liparis campylostalix* Rchb.f. | 39.72917 | 140.1042 |
| *Liparis campylostalix* Rchb.f. | 39.72917 | 141.0625 |
| *Liparis campylostalix* Rchb.f. | 39.72917 | 141.2292 |
| *Liparis campylostalix* Rchb.f. | 39.72917 | 141.3125 |
| *Liparis campylostalix* Rchb.f. | 39.77083 | 121.9792 |
| *Liparis campylostalix* Rchb.f. | 39.8125 | 140.0625 |
| *Liparis campylostalix* Rchb.f. | 39.8125 | 141.1875 |
| *Liparis campylostalix* Rchb.f. | 39.8125 | 141.4375 |
| *Liparis campylostalix* Rchb.f. | 39.85417 | 141.9792 |
| *Liparis campylostalix* Rchb.f. | 39.89583 | 139.7708 |
| *Liparis campylostalix* Rchb.f. | 39.89583 | 140.6875 |
| *Liparis campylostalix* Rchb.f. | 39.9375 | 139.8958 |
| *Liparis campylostalix* Rchb.f. | 39.97917 | 122.9792 |
| *Liparis campylostalix* Rchb.f. | 39.97917 | 140.8125 |
| *Liparis campylostalix* Rchb.f. | 39.97917 | 141.6875 |
| *Liparis campylostalix* Rchb.f. | 39.97917 | 141.7708 |
| *Liparis campylostalix* Rchb.f. | 40.02083 | 122.9792 |
| *Liparis campylostalix* Rchb.f. | 40.02083 | 124.0625 |
| *Liparis campylostalix* Rchb.f. | 40.02083 | 124.1458 |
| *Liparis campylostalix* Rchb.f. | 40.02083 | 126.3542 |
| *Liparis campylostalix* Rchb.f. | 40.02083 | 140.6042 |
| *Liparis campylostalix* Rchb.f. | 40.02083 | 141.7292 |
| *Liparis campylostalix* Rchb.f. | 40.0625 | 123.4375 |
| *Liparis campylostalix* Rchb.f. | 40.0625 | 124.0625 |
| *Liparis campylostalix* Rchb.f. | 40.10417 | 124.3958 |
| *Liparis campylostalix* Rchb.f. | 40.10417 | 140.3542 |
| *Liparis campylostalix* Rchb.f. | 40.10417 | 141.2708 |
| *Liparis campylostalix* Rchb.f. | 40.14583 | 118.3125 |
| *Liparis campylostalix* Rchb.f. | 40.14583 | 140.9375 |
| *Liparis campylostalix* Rchb.f. | 40.1875 | 140.0208 |
| *Liparis campylostalix* Rchb.f. | 40.1875 | 140.0625 |
| *Liparis campylostalix* Rchb.f. | 40.1875 | 140.4375 |
| *Liparis campylostalix* Rchb.f. | 40.1875 | 141.4375 |
| *Liparis campylostalix* Rchb.f. | 40.22917 | 124.0625 |
| *Liparis campylostalix* Rchb.f. | 40.22917 | 140.1875 |
| *Liparis campylostalix* Rchb.f. | 40.22917 | 140.7708 |
| *Liparis campylostalix* Rchb.f. | 40.22917 | 141.0208 |
| *Liparis campylostalix* Rchb.f. | 40.22917 | 141.5625 |
| *Liparis campylostalix* Rchb.f. | 40.27083 | 123.2708 |
| *Liparis campylostalix* Rchb.f. | 40.27083 | 140.5208 |
| *Liparis campylostalix* Rchb.f. | 40.27083 | 140.5625 |
| *Liparis campylostalix* Rchb.f. | 40.27083 | 140.6042 |
| *Liparis campylostalix* Rchb.f. | 40.3125 | 123.6042 |
| *Liparis campylostalix* Rchb.f. | 40.3125 | 123.7292 |
| *Liparis campylostalix* Rchb.f. | 40.3125 | 140.0208 |
| *Liparis campylostalix* Rchb.f. | 40.35417 | 122.7708 |
| *Liparis campylostalix* Rchb.f. | 40.35417 | 123.7708 |
| *Liparis campylostalix* Rchb.f. | 40.39583 | 118.9375 |
| *Liparis campylostalix* Rchb.f. | 40.39583 | 123.3125 |
| *Liparis campylostalix* Rchb.f. | 40.39583 | 123.3958 |
| *Liparis campylostalix* Rchb.f. | 40.39583 | 123.7708 |
| *Liparis campylostalix* Rchb.f. | 40.39583 | 124.0625 |
| *Liparis campylostalix* Rchb.f. | 40.39583 | 124.1042 |
| *Liparis campylostalix* Rchb.f. | 40.39583 | 139.9792 |
| *Liparis campylostalix* Rchb.f. | 40.4375 | 117.7292 |
| *Liparis campylostalix* Rchb.f. | 40.4375 | 122.6458 |
| *Liparis campylostalix* Rchb.f. | 40.4375 | 124.0625 |
| *Liparis campylostalix* Rchb.f. | 40.4375 | 141.5625 |
| *Liparis campylostalix* Rchb.f. | 40.47917 | 123.4375 |
| *Liparis campylostalix* Rchb.f. | 40.47917 | 141.6042 |
| *Liparis campylostalix* Rchb.f. | 40.52083 | 123.4792 |
| *Liparis campylostalix* Rchb.f. | 40.52083 | 141.4792 |
| *Liparis campylostalix* Rchb.f. | 40.5625 | 123.7708 |
| *Liparis campylostalix* Rchb.f. | 40.5625 | 124.0208 |
| *Liparis campylostalix* Rchb.f. | 40.64583 | 124.8125 |
| *Liparis campylostalix* Rchb.f. | 40.6875 | 125.0208 |
| *Liparis campylostalix* Rchb.f. | 40.6875 | 140.8542 |
| *Liparis campylostalix* Rchb.f. | 40.72917 | 124.7708 |
| *Liparis campylostalix* Rchb.f. | 40.77083 | 124.7292 |
| *Liparis campylostalix* Rchb.f. | 40.8125 | 118.8542 |
| *Liparis campylostalix* Rchb.f. | 40.8125 | 118.9375 |
| *Liparis campylostalix* Rchb.f. | 40.8125 | 119.9375 |
| *Liparis campylostalix* Rchb.f. | 40.8125 | 123.3958 |
| *Liparis campylostalix* Rchb.f. | 40.8125 | 124.6875 |
| *Liparis campylostalix* Rchb.f. | 40.8125 | 141.3125 |
| *Liparis campylostalix* Rchb.f. | 40.85417 | 124.6042 |
| *Liparis campylostalix* Rchb.f. | 40.85417 | 124.6458 |
| *Liparis campylostalix* Rchb.f. | 40.85417 | 124.7292 |
| *Liparis campylostalix* Rchb.f. | 40.85417 | 141.3542 |
| *Liparis campylostalix* Rchb.f. | 40.89583 | 119.1458 |
| *Liparis campylostalix* Rchb.f. | 40.89583 | 141.3542 |
| *Liparis campylostalix* Rchb.f. | 40.9375 | 123.8542 |
| *Liparis campylostalix* Rchb.f. | 40.9375 | 124.7708 |
| *Liparis campylostalix* Rchb.f. | 40.9375 | 124.8125 |
| *Liparis campylostalix* Rchb.f. | 40.9375 | 141.3125 |
| *Liparis campylostalix* Rchb.f. | 40.9375 | 141.3542 |
| *Liparis campylostalix* Rchb.f. | 40.97917 | 123.2708 |
| *Liparis campylostalix* Rchb.f. | 41.02083 | 125.8542 |
| *Liparis campylostalix* Rchb.f. | 41.10417 | 119.9375 |
| *Liparis campylostalix* Rchb.f. | 41.10417 | 123.3125 |
| *Liparis campylostalix* Rchb.f. | 41.10417 | 124.1875 |
| *Liparis campylostalix* Rchb.f. | 41.14583 | 124.1042 |
| *Liparis campylostalix* Rchb.f. | 41.14583 | 126.0208 |
| *Liparis campylostalix* Rchb.f. | 41.1875 | 124.1042 |
| *Liparis campylostalix* Rchb.f. | 41.1875 | 124.1458 |
| *Liparis campylostalix* Rchb.f. | 41.1875 | 125.7708 |
| *Liparis campylostalix* Rchb.f. | 41.22917 | 119.3958 |
| *Liparis campylostalix* Rchb.f. | 41.22917 | 119.5208 |
| *Liparis campylostalix* Rchb.f. | 41.22917 | 123.6875 |
| *Liparis campylostalix* Rchb.f. | 41.22917 | 124.1458 |
| *Liparis campylostalix* Rchb.f. | 41.22917 | 124.1875 |
| *Liparis campylostalix* Rchb.f. | 41.22917 | 125.3542 |
| *Liparis campylostalix* Rchb.f. | 41.27083 | 124.1042 |
| *Liparis campylostalix* Rchb.f. | 41.27083 | 125.3542 |
| *Liparis campylostalix* Rchb.f. | 41.27083 | 126.1458 |
| *Liparis campylostalix* Rchb.f. | 41.27083 | 126.2708 |
| *Liparis campylostalix* Rchb.f. | 41.27083 | 127.8958 |
| *Liparis campylostalix* Rchb.f. | 41.27083 | 141.1458 |
| *Liparis campylostalix* Rchb.f. | 41.3125 | 124.1042 |
| *Liparis campylostalix* Rchb.f. | 41.3125 | 124.8958 |
| *Liparis campylostalix* Rchb.f. | 41.3125 | 126.2292 |
| *Liparis campylostalix* Rchb.f. | 41.35417 | 125.3542 |
| *Liparis campylostalix* Rchb.f. | 41.39583 | 124.8125 |
| *Liparis campylostalix* Rchb.f. | 41.39583 | 124.8542 |
| *Liparis campylostalix* Rchb.f. | 41.39583 | 127.0208 |
| *Liparis campylostalix* Rchb.f. | 41.39583 | 128.1875 |
| *Liparis campylostalix* Rchb.f. | 41.4375 | 125.3125 |
| *Liparis campylostalix* Rchb.f. | 41.4375 | 127.4375 |
| *Liparis campylostalix* Rchb.f. | 41.4375 | 128.1875 |
| *Liparis campylostalix* Rchb.f. | 41.47917 | 126.6458 |
| *Liparis campylostalix* Rchb.f. | 41.47917 | 128.2708 |
| *Liparis campylostalix* Rchb.f. | 41.52083 | 126.5208 |
| *Liparis campylostalix* Rchb.f. | 41.52083 | 128.3542 |
| *Liparis campylostalix* Rchb.f. | 41.60417 | 121.6875 |
| *Liparis campylostalix* Rchb.f. | 41.6875 | 124.4792 |
| *Liparis campylostalix* Rchb.f. | 41.6875 | 125.4375 |
| *Liparis campylostalix* Rchb.f. | 41.72917 | 120.1458 |
| *Liparis campylostalix* Rchb.f. | 41.72917 | 125.0208 |
| *Liparis campylostalix* Rchb.f. | 41.72917 | 125.1875 |
| *Liparis campylostalix* Rchb.f. | 41.72917 | 125.2292 |
| *Liparis campylostalix* Rchb.f. | 41.72917 | 125.6042 |
| *Liparis campylostalix* Rchb.f. | 41.72917 | 129.6875 |
| *Liparis campylostalix* Rchb.f. | 41.8125 | 123.6042 |
| *Liparis campylostalix* Rchb.f. | 41.8125 | 124.1042 |
| *Liparis campylostalix* Rchb.f. | 41.8125 | 124.1458 |
| *Liparis campylostalix* Rchb.f. | 41.8125 | 126.8958 |
| *Liparis campylostalix* Rchb.f. | 41.85417 | 123.6042 |
| *Liparis campylostalix* Rchb.f. | 41.85417 | 126.8958 |
| *Liparis campylostalix* Rchb.f. | 41.89583 | 126.6458 |
| *Liparis campylostalix* Rchb.f. | 41.97917 | 125.2292 |
| *Liparis campylostalix* Rchb.f. | 41.97917 | 125.2708 |
| *Liparis campylostalix* Rchb.f. | 41.97917 | 125.3125 |
| *Liparis campylostalix* Rchb.f. | 42.0625 | 124.7292 |
| *Liparis campylostalix* Rchb.f. | 42.0625 | 125.8125 |
| *Liparis campylostalix* Rchb.f. | 42.10417 | 124.6875 |
| *Liparis campylostalix* Rchb.f. | 42.10417 | 124.7292 |
| *Liparis campylostalix* Rchb.f. | 42.10417 | 124.7708 |
| *Liparis campylostalix* Rchb.f. | 42.10417 | 124.9375 |
| *Liparis campylostalix* Rchb.f. | 42.10417 | 143.0208 |
| *Liparis campylostalix* Rchb.f. | 42.14583 | 125.6042 |
| *Liparis campylostalix* Rchb.f. | 42.1875 | 125.0625 |
| *Liparis campylostalix* Rchb.f. | 42.1875 | 125.8958 |
| *Liparis campylostalix* Rchb.f. | 42.1875 | 126.0208 |
| *Liparis campylostalix* Rchb.f. | 42.1875 | 126.0625 |
| *Liparis campylostalix* Rchb.f. | 42.22917 | 129.1875 |
| *Liparis campylostalix* Rchb.f. | 42.22917 | 129.2292 |
| *Liparis campylostalix* Rchb.f. | 42.22917 | 129.6875 |
| *Liparis campylostalix* Rchb.f. | 42.27083 | 125.7292 |
| *Liparis campylostalix* Rchb.f. | 42.3125 | 123.8125 |
| *Liparis campylostalix* Rchb.f. | 42.3125 | 125.3125 |
| *Liparis campylostalix* Rchb.f. | 42.3125 | 125.3542 |
| *Liparis campylostalix* Rchb.f. | 42.3125 | 127.2708 |
| *Liparis campylostalix* Rchb.f. | 42.3125 | 140.9792 |
| *Liparis campylostalix* Rchb.f. | 42.35417 | 127.2708 |
| *Liparis campylostalix* Rchb.f. | 42.39583 | 124.8958 |
| *Liparis campylostalix* Rchb.f. | 42.39583 | 125.2708 |
| *Liparis campylostalix* Rchb.f. | 42.39583 | 130.7708 |
| *Liparis campylostalix* Rchb.f. | 42.39583 | 142.3125 |
| *Liparis campylostalix* Rchb.f. | 42.4375 | 124.8958 |
| *Liparis campylostalix* Rchb.f. | 42.4375 | 128.1042 |
| *Liparis campylostalix* Rchb.f. | 42.4375 | 46.9375 |
| *Liparis campylostalix* Rchb.f. | 42.47917 | 124.8958 |
| *Liparis campylostalix* Rchb.f. | 42.47917 | 130.8542 |
| *Liparis campylostalix* Rchb.f. | 42.47917 | 130.9375 |
| *Liparis campylostalix* Rchb.f. | 42.47917 | 142.0625 |
| *Liparis campylostalix* Rchb.f. | 42.5625 | 124.8125 |
| *Liparis campylostalix* Rchb.f. | 42.5625 | 130.6875 |
| *Liparis campylostalix* Rchb.f. | 42.5625 | 142.5208 |
| *Liparis campylostalix* Rchb.f. | 42.60417 | 131.1875 |
| *Liparis campylostalix* Rchb.f. | 42.60417 | 141.3125 |
| *Liparis campylostalix* Rchb.f. | 42.60417 | 142.9792 |
| *Liparis campylostalix* Rchb.f. | 42.64583 | 130.6042 |
| *Liparis campylostalix* Rchb.f. | 42.64583 | 131.2292 |
| *Liparis campylostalix* Rchb.f. | 42.64583 | 131.4375 |
| *Liparis campylostalix* Rchb.f. | 42.64583 | 131.4792 |
| *Liparis campylostalix* Rchb.f. | 42.64583 | 142.3125 |
| *Liparis campylostalix* Rchb.f. | 42.6875 | 125.5208 |
| *Liparis campylostalix* Rchb.f. | 42.6875 | 131.3542 |
| *Liparis campylostalix* Rchb.f. | 42.6875 | 140.8958 |
| *Liparis campylostalix* Rchb.f. | 42.6875 | 141.6042 |
| *Liparis campylostalix* Rchb.f. | 42.6875 | 141.6458 |
| *Liparis campylostalix* Rchb.f. | 42.72917 | 124.7292 |
| *Liparis campylostalix* Rchb.f. | 42.72917 | 130.7708 |
| *Liparis campylostalix* Rchb.f. | 42.72917 | 131.5208 |
| *Liparis campylostalix* Rchb.f. | 42.72917 | 133.1458 |
| *Liparis campylostalix* Rchb.f. | 42.72917 | 142.3125 |
| *Liparis campylostalix* Rchb.f. | 42.77083 | 128.5208 |
| *Liparis campylostalix* Rchb.f. | 42.77083 | 132.8125 |
| *Liparis campylostalix* Rchb.f. | 42.77083 | 132.8542 |
| *Liparis campylostalix* Rchb.f. | 42.8125 | 124.9375 |
| *Liparis campylostalix* Rchb.f. | 42.8125 | 124.9792 |
| *Liparis campylostalix* Rchb.f. | 42.8125 | 130.8958 |
| *Liparis campylostalix* Rchb.f. | 42.8125 | 132.8125 |
| *Liparis campylostalix* Rchb.f. | 42.8125 | 132.9792 |
| *Liparis campylostalix* Rchb.f. | 42.8125 | 133.1042 |
| *Liparis campylostalix* Rchb.f. | 42.8125 | 142.3958 |
| *Liparis campylostalix* Rchb.f. | 42.85417 | 132.7708 |
| *Liparis campylostalix* Rchb.f. | 42.85417 | 132.8125 |
| *Liparis campylostalix* Rchb.f. | 42.85417 | 132.8542 |
| *Liparis campylostalix* Rchb.f. | 42.85417 | 132.8958 |
| *Liparis campylostalix* Rchb.f. | 42.85417 | 132.9375 |
| *Liparis campylostalix* Rchb.f. | 42.89583 | 124.3958 |
| *Liparis campylostalix* Rchb.f. | 42.89583 | 131.0625 |
| *Liparis campylostalix* Rchb.f. | 42.89583 | 131.7292 |
| *Liparis campylostalix* Rchb.f. | 42.89583 | 132.6042 |
| *Liparis campylostalix* Rchb.f. | 42.89583 | 132.7292 |
| *Liparis campylostalix* Rchb.f. | 42.9375 | 122.3542 |
| *Liparis campylostalix* Rchb.f. | 42.9375 | 124.4375 |
| *Liparis campylostalix* Rchb.f. | 42.9375 | 131.2708 |
| *Liparis campylostalix* Rchb.f. | 42.9375 | 131.7292 |
| *Liparis campylostalix* Rchb.f. | 42.9375 | 133.0625 |
| *Liparis campylostalix* Rchb.f. | 42.9375 | 133.3542 |
| *Liparis campylostalix* Rchb.f. | 42.97917 | 127.1875 |
| *Liparis campylostalix* Rchb.f. | 42.97917 | 131.7292 |
| *Liparis campylostalix* Rchb.f. | 42.97917 | 131.8542 |
| *Liparis campylostalix* Rchb.f. | 42.97917 | 131.9375 |
| *Liparis campylostalix* Rchb.f. | 42.97917 | 132.9375 |
| *Liparis campylostalix* Rchb.f. | 42.97917 | 134.1042 |
| *Liparis campylostalix* Rchb.f. | 42.97917 | 141.3958 |
| *Liparis campylostalix* Rchb.f. | 42.97917 | 143.1875 |
| *Liparis campylostalix* Rchb.f. | 42.97917 | 144.3542 |
| *Liparis campylostalix* Rchb.f. | 42.97917 | 144.9375 |
| *Liparis campylostalix* Rchb.f. | 43.02083 | 131.1458 |
| *Liparis campylostalix* Rchb.f. | 43.02083 | 131.8542 |
| *Liparis campylostalix* Rchb.f. | 43.02083 | 133.6458 |
| *Liparis campylostalix* Rchb.f. | 43.02083 | 133.7292 |
| *Liparis campylostalix* Rchb.f. | 43.02083 | 134.0208 |
| *Liparis campylostalix* Rchb.f. | 43.02083 | 134.1042 |
| *Liparis campylostalix* Rchb.f. | 43.02083 | 141.5208 |
| *Liparis campylostalix* Rchb.f. | 43.02083 | 144.4375 |
| *Liparis campylostalix* Rchb.f. | 43.0625 | 131.1458 |
| *Liparis campylostalix* Rchb.f. | 43.0625 | 131.8542 |
| *Liparis campylostalix* Rchb.f. | 43.0625 | 132.4792 |
| *Liparis campylostalix* Rchb.f. | 43.10417 | 128.8958 |
| *Liparis campylostalix* Rchb.f. | 43.10417 | 128.9375 |
| *Liparis campylostalix* Rchb.f. | 43.10417 | 131.4792 |
| *Liparis campylostalix* Rchb.f. | 43.10417 | 131.5208 |
| *Liparis campylostalix* Rchb.f. | 43.10417 | 131.5625 |
| *Liparis campylostalix* Rchb.f. | 43.10417 | 131.9792 |
| *Liparis campylostalix* Rchb.f. | 43.10417 | 132.7708 |
| *Liparis campylostalix* Rchb.f. | 43.10417 | 133.9375 |
| *Liparis campylostalix* Rchb.f. | 43.14583 | 131.9792 |
| *Liparis campylostalix* Rchb.f. | 43.14583 | 140.9375 |
| *Liparis campylostalix* Rchb.f. | 43.1875 | 129.4375 |
| *Liparis campylostalix* Rchb.f. | 43.1875 | 129.7708 |
| *Liparis campylostalix* Rchb.f. | 43.1875 | 132.6875 |
| *Liparis campylostalix* Rchb.f. | 43.1875 | 132.8958 |
| *Liparis campylostalix* Rchb.f. | 43.1875 | 133.2708 |
| *Liparis campylostalix* Rchb.f. | 43.1875 | 134.1042 |
| *Liparis campylostalix* Rchb.f. | 43.1875 | 140.8958 |
| *Liparis campylostalix* Rchb.f. | 43.1875 | 140.9375 |
| *Liparis campylostalix* Rchb.f. | 43.1875 | 145.3125 |
| *Liparis campylostalix* Rchb.f. | 43.22917 | 131.9792 |
| *Liparis campylostalix* Rchb.f. | 43.22917 | 132.0208 |
| *Liparis campylostalix* Rchb.f. | 43.22917 | 132.0625 |
| *Liparis campylostalix* Rchb.f. | 43.22917 | 132.1458 |
| *Liparis campylostalix* Rchb.f. | 43.22917 | 132.8125 |
| *Liparis campylostalix* Rchb.f. | 43.22917 | 140.8958 |
| *Liparis campylostalix* Rchb.f. | 43.22917 | 140.9792 |
| *Liparis campylostalix* Rchb.f. | 43.22917 | 142.2708 |
| *Liparis campylostalix* Rchb.f. | 43.27083 | 131.6875 |
| *Liparis campylostalix* Rchb.f. | 43.35417 | 131.3958 |
| *Liparis campylostalix* Rchb.f. | 43.35417 | 132.4375 |
| *Liparis campylostalix* Rchb.f. | 43.35417 | 133.8958 |
| *Liparis campylostalix* Rchb.f. | 43.39583 | 141.7292 |
| *Liparis campylostalix* Rchb.f. | 43.39583 | 145.7708 |
| *Liparis campylostalix* Rchb.f. | 43.4375 | 144.0625 |
| *Liparis campylostalix* Rchb.f. | 43.4375 | 145.9375 |
| *Liparis campylostalix* Rchb.f. | 43.47917 | 124.8125 |
| *Liparis campylostalix* Rchb.f. | 43.47917 | 131.7292 |
| *Liparis campylostalix* Rchb.f. | 43.47917 | 131.9792 |
| *Liparis campylostalix* Rchb.f. | 43.47917 | 141.6875 |
| *Liparis campylostalix* Rchb.f. | 43.5625 | 132.3125 |
| *Liparis campylostalix* Rchb.f. | 43.5625 | 134.3958 |
| *Liparis campylostalix* Rchb.f. | 43.5625 | 134.4375 |
| *Liparis campylostalix* Rchb.f. | 43.5625 | 144.1458 |
| *Liparis campylostalix* Rchb.f. | 43.60417 | 131.4375 |
| *Liparis campylostalix* Rchb.f. | 43.60417 | 142.6458 |
| *Liparis campylostalix* Rchb.f. | 43.60417 | 144.3125 |
| *Liparis campylostalix* Rchb.f. | 43.60417 | 144.3542 |
| *Liparis campylostalix* Rchb.f. | 43.60417 | 144.4375 |
| *Liparis campylostalix* Rchb.f. | 43.64583 | 132.3125 |
| *Liparis campylostalix* Rchb.f. | 43.64583 | 132.4792 |
| *Liparis campylostalix* Rchb.f. | 43.64583 | 132.5208 |
| *Liparis campylostalix* Rchb.f. | 43.64583 | 143.4375 |
| *Liparis campylostalix* Rchb.f. | 43.64583 | 143.9792 |
| *Liparis campylostalix* Rchb.f. | 43.64583 | 146.3542 |
| *Liparis campylostalix* Rchb.f. | 43.6875 | 127.1458 |
| *Liparis campylostalix* Rchb.f. | 43.6875 | 127.3958 |
| *Liparis campylostalix* Rchb.f. | 43.6875 | 132.3958 |
| *Liparis campylostalix* Rchb.f. | 43.6875 | 134.1875 |
| *Liparis campylostalix* Rchb.f. | 43.6875 | 142.3958 |
| *Liparis campylostalix* Rchb.f. | 43.6875 | 142.4375 |
| *Liparis campylostalix* Rchb.f. | 43.6875 | 143.6458 |
| *Liparis campylostalix* Rchb.f. | 43.72917 | 132.3958 |
| *Liparis campylostalix* Rchb.f. | 43.72917 | 134.4375 |
| *Liparis campylostalix* Rchb.f. | 43.77083 | 128.4375 |
| *Liparis campylostalix* Rchb.f. | 43.77083 | 131.9375 |
| *Liparis campylostalix* Rchb.f. | 43.77083 | 142.3542 |
| *Liparis campylostalix* Rchb.f. | 43.8125 | 125.4792 |
| *Liparis campylostalix* Rchb.f. | 43.8125 | 127.9792 |
| *Liparis campylostalix* Rchb.f. | 43.8125 | 131.1875 |
| *Liparis campylostalix* Rchb.f. | 43.8125 | 143.8542 |
| *Liparis campylostalix* Rchb.f. | 43.8125 | 144.2292 |
| *Liparis campylostalix* Rchb.f. | 43.85417 | 127.6458 |
| *Liparis campylostalix* Rchb.f. | 43.85417 | 143.8542 |
| *Liparis campylostalix* Rchb.f. | 43.85417 | 143.8958 |
| *Liparis campylostalix* Rchb.f. | 43.85417 | 144.1042 |
| *Liparis campylostalix* Rchb.f. | 43.85417 | 145.5208 |
| *Liparis campylostalix* Rchb.f. | 43.89583 | 131.5625 |
| *Liparis campylostalix* Rchb.f. | 43.89583 | 135.2292 |
| *Liparis campylostalix* Rchb.f. | 43.89583 | 143.8958 |
| *Liparis campylostalix* Rchb.f. | 43.89583 | 145.6458 |
| *Liparis campylostalix* Rchb.f. | 43.9375 | 125.8542 |
| *Liparis campylostalix* Rchb.f. | 43.9375 | 131.3958 |
| *Liparis campylostalix* Rchb.f. | 43.9375 | 131.4792 |
| *Liparis campylostalix* Rchb.f. | 43.9375 | 131.6042 |
| *Liparis campylostalix* Rchb.f. | 43.9375 | 135.4375 |
| *Liparis campylostalix* Rchb.f. | 43.9375 | 143.7292 |
| *Liparis campylostalix* Rchb.f. | 43.9375 | 144.3958 |
| *Liparis campylostalix* Rchb.f. | 43.97917 | 132.2292 |
| *Liparis campylostalix* Rchb.f. | 43.97917 | 144.0625 |
| *Liparis campylostalix* Rchb.f. | 44.02083 | 131.3958 |
| *Liparis campylostalix* Rchb.f. | 44.02083 | 145.8542 |
| *Liparis campylostalix* Rchb.f. | 44.0625 | 131.3958 |
| *Liparis campylostalix* Rchb.f. | 44.10417 | 145.1042 |
| *Liparis campylostalix* Rchb.f. | 44.14583 | 132.9792 |
| *Liparis campylostalix* Rchb.f. | 44.14583 | 133.7708 |
| *Liparis campylostalix* Rchb.f. | 44.1875 | 135.3958 |
| *Liparis campylostalix* Rchb.f. | 44.1875 | 135.6042 |
| *Liparis campylostalix* Rchb.f. | 44.1875 | 135.6458 |
| *Liparis campylostalix* Rchb.f. | 44.1875 | 145.9375 |
| *Liparis campylostalix* Rchb.f. | 44.22917 | 135.0625 |
| *Liparis campylostalix* Rchb.f. | 44.22917 | 135.3542 |
| *Liparis campylostalix* Rchb.f. | 44.27083 | 135.0625 |
| *Liparis campylostalix* Rchb.f. | 44.3125 | 131.0625 |
| *Liparis campylostalix* Rchb.f. | 44.35417 | 129.4792 |
| *Liparis campylostalix* Rchb.f. | 44.35417 | 143.3542 |
| *Liparis campylostalix* Rchb.f. | 44.39583 | 132.3125 |
| *Liparis campylostalix* Rchb.f. | 44.39583 | 135.3958 |
| *Liparis campylostalix* Rchb.f. | 44.39583 | 146.3958 |
| *Liparis campylostalix* Rchb.f. | 44.47917 | 135.4375 |
| *Liparis campylostalix* Rchb.f. | 44.52083 | 132.6458 |
| *Liparis campylostalix* Rchb.f. | 44.52083 | 142.7292 |
| *Liparis campylostalix* Rchb.f. | 44.5625 | 135.5625 |
| *Liparis campylostalix* Rchb.f. | 44.5625 | 135.6042 |
| *Liparis campylostalix* Rchb.f. | 44.5625 | 135.9375 |
| *Liparis campylostalix* Rchb.f. | 44.64583 | 130.0625 |
| *Liparis campylostalix* Rchb.f. | 44.64583 | 132.6042 |
| *Liparis campylostalix* Rchb.f. | 44.72917 | 136.1042 |
| *Liparis campylostalix* Rchb.f. | 44.72917 | 142.2292 |
| *Liparis campylostalix* Rchb.f. | 44.89583 | 135.8542 |
| *Liparis campylostalix* Rchb.f. | 44.89583 | 136.5208 |
| *Liparis campylostalix* Rchb.f. | 44.97917 | 136.5625 |
| *Liparis campylostalix* Rchb.f. | 45.0625 | 136.6875 |
| *Liparis campylostalix* Rchb.f. | 45.10417 | 133.3542 |
| *Liparis campylostalix* Rchb.f. | 45.10417 | 136.3125 |
| *Liparis campylostalix* Rchb.f. | 45.14583 | 136.3125 |
| *Liparis campylostalix* Rchb.f. | 45.22917 | 127.9375 |
| *Liparis campylostalix* Rchb.f. | 45.27083 | 136.1458 |
| *Liparis campylostalix* Rchb.f. | 45.3125 | 136.8125 |
| *Liparis campylostalix* Rchb.f. | 45.3125 | 141.0208 |
| *Liparis campylostalix* Rchb.f. | 45.77083 | 132.9792 |
| *Liparis campylostalix* Rchb.f. | 45.77083 | 135.4792 |
| *Liparis campylostalix* Rchb.f. | 46.22917 | 141.2292 |
| *Liparis campylostalix* Rchb.f. | 46.35417 | 141.6042 |
| *Liparis campylostalix* Rchb.f. | 46.64583 | 142.7708 |
| *Liparis campylostalix* Rchb.f. | 46.6875 | 134.7708 |
| *Liparis campylostalix* Rchb.f. | 46.6875 | 135.7708 |
| *Liparis campylostalix* Rchb.f. | 46.72917 | 131.1458 |
| *Liparis campylostalix* Rchb.f. | 46.8125 | 134.0208 |
| *Liparis campylostalix* Rchb.f. | 47.3125 | 136.5208 |
| *Liparis campylostalix* Rchb.f. | 47.77083 | 128.8958 |
| *Liparis campylostalix* Rchb.f. | 47.9375 | 136.0208 |
| *Liparis campylostalix* Rchb.f. | 48.02083 | 134.9375 |
| *Liparis campylostalix* Rchb.f. | 48.35417 | 135.5625 |
| *Liparis campylostalix* Rchb.f. | 48.60417 | 135.5208 |
| *Liparis campylostalix* Rchb.f. | 48.89583 | 130.6458 |
| *Liparis campylostalix* Rchb.f. | 49.02083 | 133.0625 |
| *Liparis campylostalix* Rchb.f. | 49.0625 | 130.5625 |
| *Liparis campylostalix* Rchb.f. | 49.22917 | 130.6458 |
| *Liparis campylostalix* Rchb.f. | 49.3125 | 130.5625 |
| *Liparis campylostalix* Rchb.f. | 49.4375 | 129.6458 |
| *Liparis campylostalix* Rchb.f. | 49.5625 | 130.1042 |
| *Liparis campylostalix* Rchb.f. | 49.9375 | 127.6042 |
| *Liparis campylostalix* Rchb.f. | 50.10417 | 129.9792 |
| *Liparis campylostalix* Rchb.f. | 50.3125 | 127.4792 |
| *Liparis campylostalix* Rchb.f. | 50.52083 | 137.2708 |
| *Liparis campylostalix* Rchb.f. | 50.5625 | 137.6875 |
| *Liparis campylostalix* Rchb.f. | 50.77083 | 137.5208 |
| *Liparis campylostalix* Rchb.f. | 50.77083 | 137.6458 |
| *Liparis campylostalix* Rchb.f. | 50.8125 | 137.2292 |
| *Liparis campylostalix* Rchb.f. | 52.8125 | 130.3542 |
| *Liparis campylostalix* Rchb.f. | 53.22917 | 93.02083 |
| *Liparis campylostalix* Rchb.f. | 54.77083 | 158.7708 |
